# Exploratory analysis of long-term suppressive therapy with dalbavancin in ventricular assist device infections caused by *Staphylococcus aureus*

**DOI:** 10.1038/s41598-025-99112-7

**Published:** 2025-05-03

**Authors:** Benedict Morath, Sabrina Klein, Ute Chiriac, Yvonne Müller, Lisa Koeppel, Otto Frey, Heike Lanzinger, Philipp Schlegel, Sonja Hamed, Dennis Nurjadi, Philipp Ehlermann, Matthias Karck, Anna L. Meyer

**Affiliations:** 1 Hospital Pharmacy, Heidelberg University Hospital, Im Neuenheimer Feld 670, Heidelberg, Germany; 2 Department of Infectious Diseases, Medical Microbiology and Hygiene, Medical Faculty Heidelberg, Heidelberg University, Im Neuenheimer Feld 324, 69120 Heidelberg, Germany; 3 Department of Infectious Diseases, Medical Microbiology and Hygiene, Heidelberg University Hospital, Im Neuenheimer Feld 324, 69120 Heidelberg, Germany; 4 Department of Cardiac Surgery, Heidelberg University Hospital, Im Neuenheimer Feld 420, 69120 Heidelberg, Germany; 5 Department of Infectious Disease and Tropical Medicine, Heidelberg University, Im Neuenheimer Feld 324, 69120 Heidelberg, Germany; 6 Hospital Pharmacy, General Hospital Heidenheim, 89522 Heidenheim, Germany; 7 Department of Cardiology, Heidelberg University Hospital, Im Neuenheimer Feld 410, 69120 Heidelberg, Germany; 8 Department of Infectious Diseases and Microbiology, University of Lübeck and University Medical Center Schleswig-Holstein Campus Lübeck, Lübeck, Germany; 9 German Center for Infection Research (DZIF), Partner Site Hamburg-Lübeck-Borstel-Riems, Lübeck, Germany

**Keywords:** Dalbavancin, Ventricular assist device, *Staphylococcus aureus*, Persistent infection, Drug safety, Bacterial infection, Cardiomyopathies

## Abstract

To analyze dalbavancin’s potential for long-term suppressive therapy in patients with infected ventricular assist devices (VAD), the VAD register of Heidelberg University Hospital was searched for patients who received dalbavancin for long-term suppression therapy. Clinical data, laboratory, and microbiological results were extracted. Healthcare utilization was analyzed by number of hospitalizations before and during dalbavancin therapy with a generalized linear mixed model. Drug safety was investigated with regard to liver and renal function, and thrombocyte count. Thirteen patients were included in the study; receiving a regimen of 1500 mg dalbavancin at day 1 and day 8 with repetition of the cycle at day 42. The mean follow-up time was 254 days (IQR 252). Eight patients were treated for driveline infection and five patients for driveline and blood stream infection, all of them caused by *Staphylococcus aureus*. The majority of patients (n = 11/13) underwent surgical wound debridement and intravenous antibiotic therapy before start of dalbavancin. Under dalbavancin therapy, no blood stream infection was observed and significantly fewer hospitalizations occurred with an odds ratio of 0.27 (*p* < 0.001). In four patients, elevations of liver transaminases were detected and led to discontinuation of dalbavancin therapy in one patient.

**Supplementary Information:**

The online version contains supplementary material available at 10.1038/s41598-025-99112-7.

## Introduction

The treatment of persistent or recurrent infections caused by *Staphylococcus aureus* remains challenging. In outpatient settings, therapeutic options for oral antimicrobial therapy are often limited due to several aspects. Beside the risk of inadequate exposure when orally administered^1^, antibiotics with a good oral bioavailability frequently have a higher risk of adverse drug events (ADE), have black box warnings for special patient populations, and might aggravate management due to drug–drug interactions^2–5^.

Dalbavancin is a lipoglycopeptide antibiotic approved for acute bacterial and skin and soft tissue infections with an antimicrobial spectrum against Gram-positive pathogens (e.g. methicillin-susceptible and methicillin-resistant *S. aureus*, *Streptococcus* spp*.*) and provides an alternative strategy in these situations^6^. Dalbavancin has a reported terminal half-life of 372 h allowing an intravenous single dose of 1500 mg to cover a period of at least 2 weeks^7^. Besides the indicated dosages, repetitive dosages (e.g. 1500 mg on day 1 and 8) to cover periods of 6 weeks have been investigated and showed promising effects^8,9^. Moreover, case reports suggest the potential for long-term suppression therapy and expert opinions on recommended dosage have been issued^8,10^.

In patients with terminal heart failure, implantation of ventricular assist devices (VAD) often is the last therapeutic option^11,12^. Unfortunately, device-associated infections are a common and severe complication being the underlying reason in up to 15% of deceased patients^12,13^. Gram-positive bacteria, in particular *Staphylococcus* spp*.*, are the dominant causal pathogen, although Gram-negative infections are observed^13,14^. Therapeutic strategies in driveline-associated infections include surgical wound debridement, vacuum-assisted closure (VAC) therapy accompanied by targeted intravenous antimicrobial therapy, often with subsequent oral suppression therapy. Nevertheless, relapse infection rates appear to be high^15,16^.

However, there is sparse information on dalbavancin long-term use in patients with VAD-infections and how dalbavancin long-term therapy could be implemented into routine care also with regard to drug safety and monitoring^17,18^. Therefore, VAD-patients on long-term suppression with dalbavancin within a large tertiary care center were analyzed in this study.

## Methods

The study followed the ‘Strengthening the reporting of observational studies in epidemiology’ (STROBE)-statement^19^. All methods were performed in accordance with the relevant guidelines and regulations and the study was approved by the Heidelberg Ethics Committee (S-674/2022). All participants and/or their legal guardian(s) included in the Heidelberg register for VAD infection provided informed consent.

### Setting and procedures

#### Antibiotic therapy

The department of cardiac surgery and the department of cardiology care for around 82 outpatient VAD patients. Following intravenous antibiotic therapy and surgical debridement, patients with recurrent driveline infection (DLI) and/or concomitant blood stream infection (BSI) receive long-term suppressive antibiotic therapy depending on antibiotic susceptibility testing, co-medication, and tolerability. In patients with therapeutic failure under, or contraindications for antibiotic substances appropriate for oral suppression therapy, dalbavancin is started as last line of therapy. Patients on dalbavancin treatment received regular laboratory monitoring and measurement of serum drug concentrations^20^.

#### Microbiologic diagnostics

Microbiological diagnostic was routinely performed on tissue specimen or swabs from the driveline and blood cultures when clinically indicated. Routine screening for *S. aureus* (MSSA) and multidrug resistant organisms (MDRO; including Vancomycin-resistant *Enterococcus* spp. (VRE), Methicillin-resistant *S. aureus* (MRSA) and carbapenem-resistant *Enterobacterales*, *P. aeruginosa* and *Acinetobacter baumannii complex*) were obtained before start of and during dalbavancin therapy. Infection isolates were tested and interpreted following EUCAST standards and breakpoints valid in the respective year^21^. Determination of minimal inhibitory concentration (MIC) of dalbavancin was performed with MIC strips test (Ref. 92137 Liofilchem, Italy) and microdilution assay according to EUCAST standards and manufacturer’s instructions^21^.

### Data collection

The register of patients with a VAD-associated infection was queried for all patients on dalbavancin long-term suppression therapy. Patients were eligible for analysis if they had received at least two off-label doses of 1500 mg dalbavancin. The following data were extracted: Age, sex, comorbidities, date of VAD implantation, and hospitalizations before and after dalbavancin start, dalbavancin administration dates and dosages.

In addition, serum creatinine, estimated glomerular filtration rate (eGFR) according to chronic kidney disease epidemiology collaboration (CKD-Epi) function, glutamate-oxalacetate transferase (GOT), glutamate-pyruvate transferase (GPT), alkaline phosphatase (AP), gamma-glutamine transferase (GGT), serum bilirubin, c-reactive protein (CRP), international normalized ratio (INR), leucocyte, and thrombocyte count were extracted. Results of microbiological diagnostics from tissue specimen and wound swabs, blood cultures, and from MDRO screening as well as adjusted wound score and wound status according to DESTINE were extracted from the register^22^. Due to the retrospective analysis, a comparator or control group was not available from the register, as patients with the same device and comparable characteristics and infections were all treated with dalbavancin as by clinical decision and not following a study protocol. Instead, we decided to compare infection-related hospitalizations before and under dalbavancin therapy in each individual patient.

### Data analysis

Data was analyzed for the period from the first dose of dalbavancin to 30th June 2023. The data analysis started from VAD implantation and ended with stopping dalbavancin therapy.

#### Clinical outcomes

##### Outcome

Outcome was stratified in the categories therapy ongoing, successful heart transplantation, therapy stopped, and therapy ended. For intentionally stopped therapies, reasons for stopping were documented.

##### Hospital utilization

Number of infection related hospitalizations, i.e. BSI or due to VAD infection were analyzed before and under therapy with dalbavancin.

##### In-hospital days under dalbavancin

The number of in-hospital days due to VAD-infection before and after start of dalbavancin was analyzed on individual patient level using, a generalized linear mixed model (GLMM).

#### Microbiology

Results from microbiological diagnostics from driveline specimen and blood cultures were analyzed including colonization with MDRO and MSSA.

#### Safety

##### Liver function

Potential elevations were rated in relation to upper limit of normal (ULN) according to local laboratory reference. For GOT and GPT 150 units per liter (U/L) was rated as 3 × ULN and 250 U/L as 5 × ULN. GGT was rated as 3 × ULN with 180 U/L and 5 × ULN at 300 U/L. The ULN for total bilirubin was defined as 1 mg/L.

##### Renal function

Renal function was analyzed according to changes in stages for patients with chronic kidney disease^23^. A potential decline in renal function was assessed by cardiologists and cardiac surgeons for other non-dalbavancin related reason, e.g. hemodynamic status.

##### INR

Changes in INR and phenprocoumon dosage under dalbavancin were investigated for potential interference of dalbavancin with phenprocoumon. Therefore, the proportion of outpatient INR measurements in therapeutic range was analyzed.

#### Statistics

Descriptive statistics were reported using means and standard deviations when appropriate. For patient level analysis of hospital utilization, a GLMM was utilized to adjust for the small sample size. A binominal response modelled the number of hospital days (successes) amongst the number of total days per patient. A fixed effect was included to discern the time before and after the intervention and a random effect for each patient was added to account for variability in the individual response. The model was fitted using maximum likelihood methodology. The estimated coefficient of the fixed effect was exponentiated to obtain an odds ratio (OR) and the according *p* value was calculated using the Wald test. Model analyses were performed using the R software environment (R Foundation for Statistical Computing, Vienna, Austria) version 4.0.4. Visualizations were performed using GraphPad Prism, version 9.5.1. (San Diego, California).

## Results

Of 167 screened patients, 13 patients were eligible for analysis. All patients were male with an implanted Heart Mate III (Abbott) (Table 1).

**Table 1:** Demographic data of the patient population.

Category	Status at start of dalbavancin
Population	
Sex	
Male (n)	13/13
Mean age (years)	57 ± 9
Active smoking status (n)	2/13
Left ventricular assist device	
Abbott heart mate 3 (n)	13/13
Mean days on ventricular assist device support (days)	719 ± 452
Diabetes mellitus type II (n)	5/13
Dialysis	0
Non-ischemic cardiomyopathy (n)	12/13
Ischemic cardiomyopathy (n)	1/13
Coronary artery diseases (n)	4/13
Therapeutic aim	
Listed for transplantation (n)	7/13
Destination therapy (n)	2/13
Evaluation for listing ongoing (n)	4/13

VAD were implanted between 2017 and 2021 and the mean time from implantation to start of dalbavancin was 719 ± 452 days. The patients had a median of 1 (IQR 2) hospitalizations for intravenous antibiotic therapy and surgical wound debridement before start of dalbavancin. The majority of patients were listed for or under evaluation for heart transplantation, whereas for two patients VAD were implanted as destination therapy.

Before start of dalbavancin therapy, eleven patients underwent surgical wound debridement with concomitant intravenous antibiotic therapy targeted for the causative pathogen. Two patients (B, I) did not receive surgical source control before start of dalbavancin.

At start of dalbavancin therapy, leucocytes, and CRP were low and other relevant laboratory parameters (renal function, INR) were in target range (Table 2). For a detailed list on diagnoses of each patient, please see Supplementary Information Table SI1.

**Table 2:** Laboratory parameters and microbiology findings at baseline.

Laboratory parameters	Measured at start of dalbavancin
eGFR (mL/min/1.73 m^2^)	78.0 ± 26.3
C-reactive protein (mg/L)	7.4 ± 5.7
Leucocytes (/nL)	8.3 ± 1.0
Thrombocytes (/nL)	238.4 ± 72.0
INR	2.3 ± 0.4
Infection site	
Driveline only	8
Driveline and BSI	5
Microbiology	
*Staphylococcus aureus* (MSSA)	12/13
MRSA	1/13

*BSI* blood stream infection, *eGFR* estimated glomerular filtration rate, *INR* international normalized ratio, *MSSA* methicillin-susceptible *Staphylococcus aureus*, *MRSA* methicillin-resistant *Staphylococcus aureus.*

### Therapy and dosing regimes

**Fig. 1:**
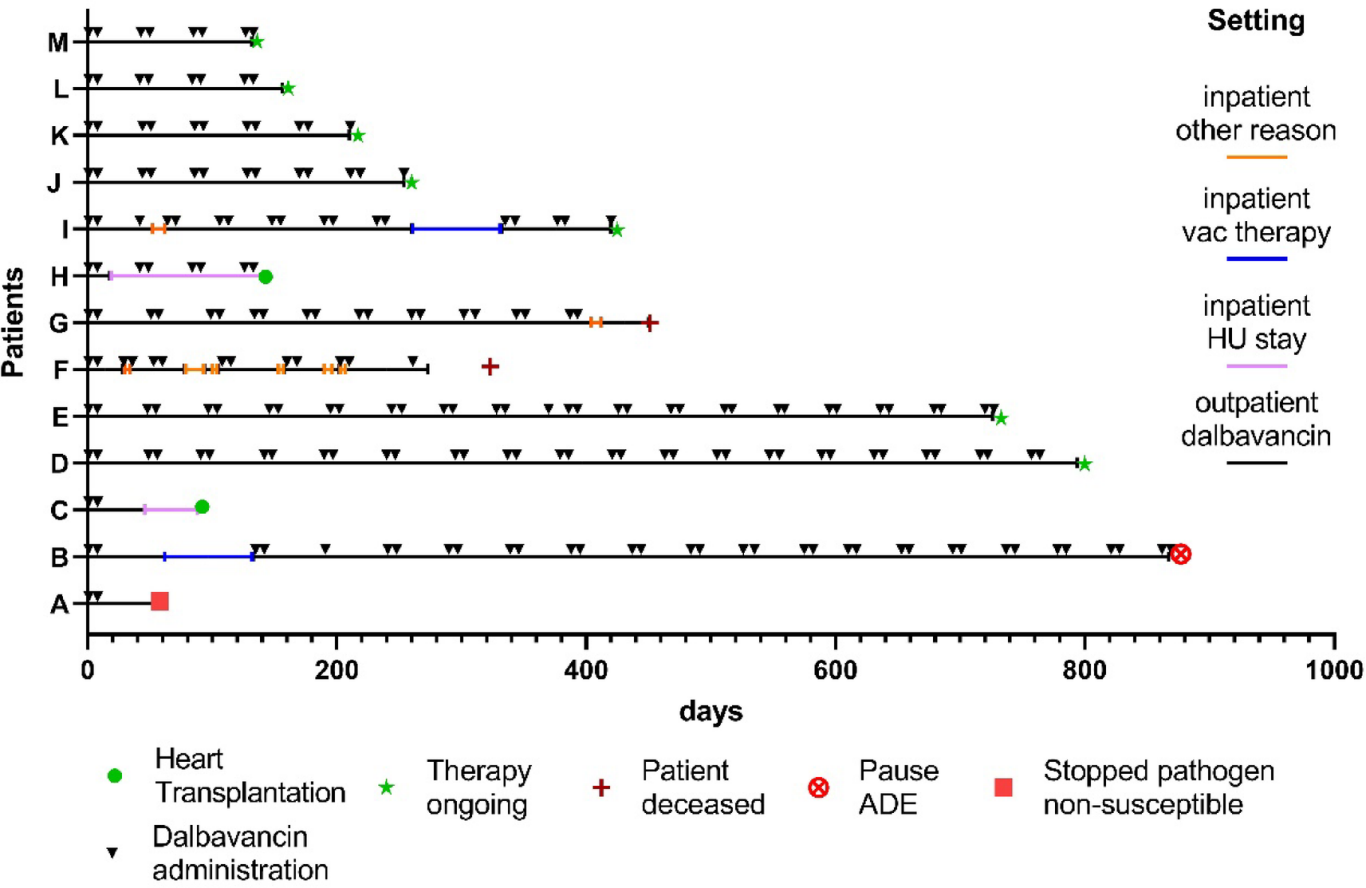
Dalbavancin administrations, hospitalizations, and outcomes under dalbavancin long-term suppression therapy. *ADE* adverse drug events, *HU* high urgency.

All patients received a regimen of repetitive dalbavancin doses of 1500 mg at day 1 and 1500 mg at day 8. The cycles were repeated at day 42 (Fig. 1). Results and implications of population pharmacokinetic analyses have been published elsewhere^24^.

### Clinical outcomes

#### Outcome and parameters

At time of data extraction, seven patients had ongoing dalbavancin therapy, two patients had received heart transplantation (Fig. 1). Therapy was stopped in patient B due to a possible ADE (increase in liver transaminases), in patient A due to detection of *Klebsiella pneumoniae,* and in patient F as the therapy goal changed to be palliative. Two patients (F and G) died because of pulmonary and cardiac decompensation (patient F) and right ventricular failure (patient G).

Following initial elevation, CRP and leucocyte count were mostly in reference range and—if elevated—not rated as clinically relevant (individual CRP and leucocyte courses are shown in detail in the Supplementary Information). Wound score and wound status dropped after initial elevation and showed no increase during the therapeutic course (Supplementary Information Fig. SI1) except for patient I prior to hospitalization for VAC-therapy (Supplementary Information Fig. SI17).

#### Hospital utilization

Before start of dalbavancin, 29 infection-related hospitalizations occurred (five due to BSI) (Table 3).

**Table 3:** Causes and durations of infection associated hospitalizations before and under dalbavancin therapy.

	Before dalbavancin	Under dalbavancin therapy
Total days	9347	4474
Hospitalizations for driveline infection (n)	24	2
Hospitalizations for BSI (n)	5	0
In-hospital days for infection associated therapy (n (%))	860 (9.2)	147 (3.3)

There were twelve hospitalizations during dalbavancin therapy, eight because of cardiac decompensation (patients F, G, I) and two for high-urgency listing for heart transplantation (patient C, H) (Fig. 1). Two patients without initial wound debridement and intravenous therapy prior to the dalbavancin course, were readmitted for VAC-therapy (B, I) (Table 3). Under dalbavancin therapy, none of the patients was hospitalized due to BSI. An approximately 73% decrease in the odds of a hospitalization (OR 0.27 (*p* < 0.001)) was observed in the GLMM (for individual hospitalization days see Supplementary Table SI2.

### Infection and microbiology

All patients were treated either for recurrent and/or persistent DLI (n = 8) or DLI associated with BSI (n = 5) caused by *S. aureus*. One isolate was MRSA (patient E). All infection isolates were susceptible to dalbavancin according to EUCAST breakpoints and MIC ranged between 0.032 and 0.125 mg/L. Under dalbavancin therapy, no BSI was observed.

Following surgical wound debridement with concomitant antibiotic therapy before start of dalbavancin, *S. aureus* was not detected in any of the specimen of the drivelines anymore.

Nevertheless, a variety of microorganisms was observed in specimen from the drivelines. In four patients (A, B, H and I), the therapy was adopted due to clinical relevance of the culture results.

In patient A, cultivation of *Serratia marcescens* resulted in change of the antibiotic therapy and discontinuation of dalbavancin. From the driveline of patient B, *S. aureus* was cultured until day 58 of dalbavancin therapy; following surgical source control and concomitant intravenous antibiotic therapy with cefazolin and rifampicin, *S. aureus* was not cultured anymore from the driveline. Cultivation of different bacterial species later on did not alter antibiotic therapy. *Klebsiella pneumoniae*, *S. haemolyticus,* and *S. epidermidis* were cultured from the driveline of patient H. This led to surgical intervention and addition of ampicillin/sulbactam while dalbavancin therapy was continued.* Staphylococcus aureus* was cultured from the driveline of patient I over a period of 279 days until surgical source control was performed accompanied by intravenous antibiotic therapy (flucloxacillin with fosfomycin followed by cefazolin and fosfomycin); thereafter, *S. haemolyticus* und *S. epidermidis* were cultured from the driveline under dalbavancin therapy and judged as being irrelevant. In patients C, F and M, all cultures from the driveline were sterile.

In five patients (A, B, C, F, I), colonisation with MSSA was observed; four with nasal and one with rectal colonisation (B). Rectal colonisation with VRE was detected in three patients (H, K, L). Patient K was already colonized with VRE before start of dalbavancin, VRE colonization was documented in patient L at time of the first dose of dalbavancin, and in patient H, VRE was first detected on day 19 after therapy initiation. No colonization with MRSA or Carbapenem-resistant Gram-negative Enterobacterales, *Pseudomonas aeruginosa* or *Acinetobacter baumanni* was detected.

A detailed overview over microbiological findings under dalbavancin therapy is provided in the Supplementary Information Table SI3.

### Safety

#### Liver function

**Fig. 2:**
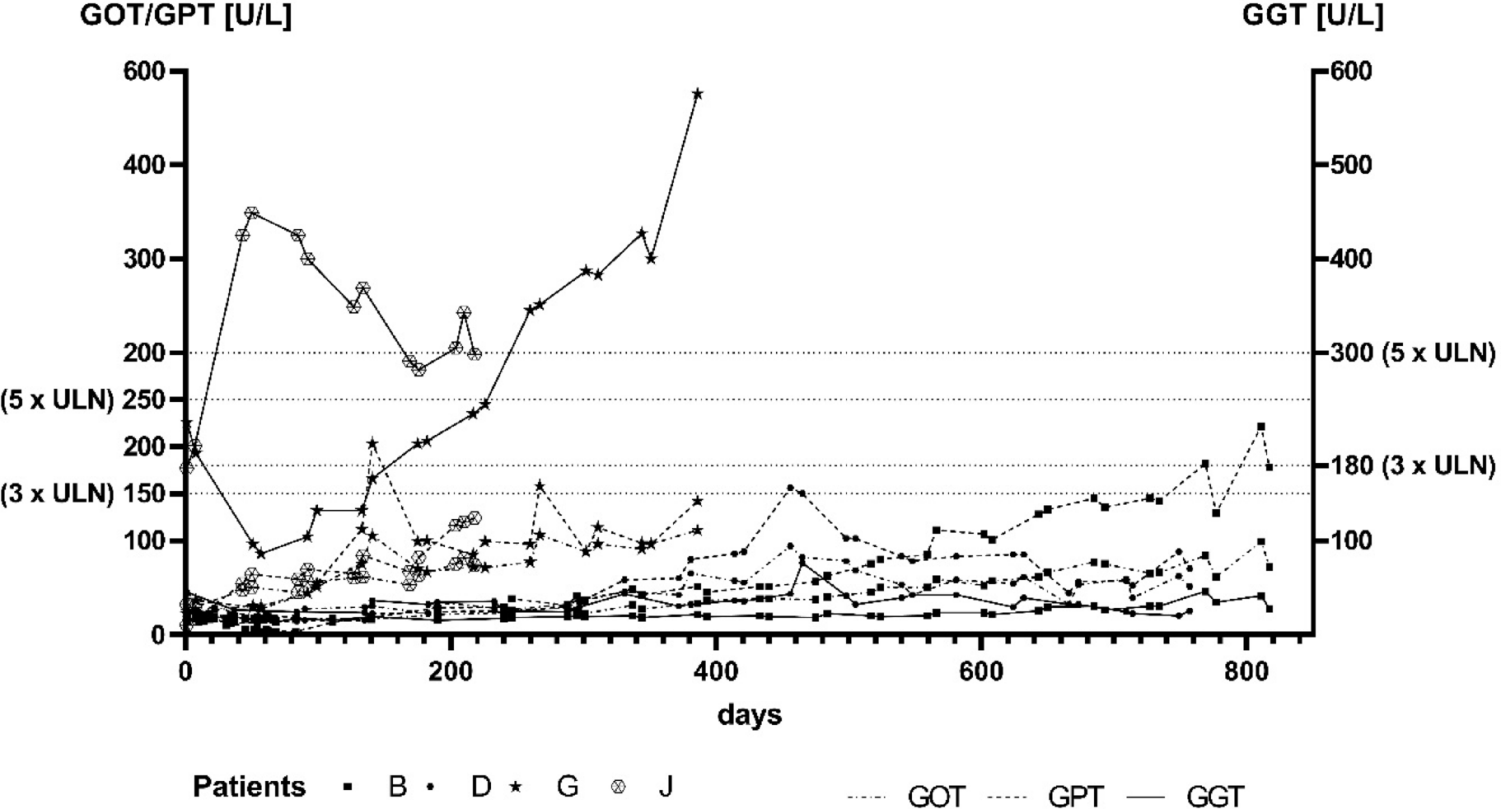
Liver transaminases values during dalbavancin therapy of patients B, D, G, J. *GOT* glutamate-oxalacetate transferase, *GPT* glutamate-pyruvate transferase, *GGT* gamma-glutamine transferase, *ULN* upper limit of normal.

In total, four patients showed elevations in liver parameters. GGT was elevated > 5 × ULN in patient J and G with concomitant but smaller raises in GOT and GPT (mostly < 3 × ULN) (Fig. 2).

These elevations were rated as sequelae of an impaired right ventricular function of which patient G deceased. In patient B, GOT and GPT continuously increased over a course of 868 days, and dalbavancin was stopped at GPT > 3 × ULN. Laboratory values returned to normal after discontinuation. No hospitalization or relapse have occurred since the therapy was stopped. In addition, patient D showed a small GPT elevation (< 2 × ULN) that was frequently monitored under ongoing therapy (day 765). None of the patients had increased bilirubin or showed other symptoms of liver injury. GPT, GOT, and GGT values of all patients are available in the Supplementary Information Figs. SI3–SI26.

#### Renal function

No dalbavancin associated decrease in renal function was observed in any of the patients (Supplementary Information Tables SI4–SI16). Other reasons for small or temporary declines in renal function included diarrhea, excessive diuresis, non-adherence to medication, and progression of heart failure (i.e. right heart failure).

#### INR

Of 212 INR measurements performed during out- and inpatient visits, 85.4% of measurements were in the therapeutic range (INR 2.0–3.0, INR 1.7–2.0 for patient F). No changes in dosages due to dalbavancin were reported by the patients (Supplementary Information Fig. SI2).

## Discussion

We report a cohort of 13 LVAD patients that received dalbavancin as long-term suppression therapy for persistent and/or recurrent device and driveline infections. Overall, treatment with dalbavancin significantly reduced the odds of hospitalizations in these patients compared to previous approaches (OR 0.27 (*p* < 0.001)) in the exploratory analyses. No BSI was observed under dalbavancin therapy. Seven patients were still undergoing long-term prophylaxis and two patients received heart transplantation. Two patients died unrelated to therapy and therapy was stopped in two patients (unsusceptible pathogen, potential ADE).

The successful application of dalbavancin in persistent VAD infections has been demonstrated in few case reports and studies^17,18,25^. Indeed, following surgical source control and concomitant intravenous therapy, we did not observe any breakthrough infection in our cohort. A recent study by Rowe et al. reported breakthrough infections in five of eight patients between 1 and 12 months after therapy initiation, although high doses of up to 1500 mg biweekly were used^18^. In our cohort, two patients were hospitalized for surgical VAC therapy. These two patients had no surgical source control before therapy initiation and driveline cultures kept on growing *S. aureus* until surgical intervention. In patients with initial wound debridement, no *S. aureus* in specimen from the driveline was observed. Together with the observations by Rowe et al., these results suggest that initial wound debridement as source control may be necessary and cannot be replaced by sole antibiotic therapy, even with higher doses of dalbavancin.

Within our cohort, dosing frequency was lower compared to other studies and consisted of repetitive 6 week cycles of two administrations of 1500 mg dalbavancin (day 1, day 8) providing adequate exposure as determined by measurements of serum drug concentration^20^. This regimen was successfully administered in a previous phase II-trial for osteomyelitis and a recent expert opinion recommends two administrations of 1500 mg within 4 weeks to cover a period of up to 6 weeks^8,9^. Moreover, a population-pharmacokinetic analyses of this population showed that also other dosing regimen guided by therapeutic drug monitoring e.g. 1500 mg q 4–5 weeks could be applied^24^. In a different population consisting of mainly patients being treated for endocarditis or prosthetic joint infections, data even support the extension of dosing cycles to 6 or 8 weeks, although protein binding was no assessed^26^. A higher protein binding up to 99% than the protein binding of 93% stated in the label is reported^27^ and the protein binding in this cohort varied between 96 and 98%^24,26^*.* For determination of cycle duration, the pathogen’s MIC and the individual dalbavancin protein binding of the patient should be considered. Thereby, overall use of dalbavancin and consequently cost can be reduced during long-term outpatient parenteral therapy.

During long-term therapies, ADE monitoring is of particular importance as some ADE occur due to chronic use or are concentration-dependent e.g. related to undetected accumulation. In this study, four patients showed mild to significant elevations in serum transaminases (GPT > GOT), that led to the withdrawal of dalbavancin in one patient when GPT was 3 × ULN. The incidence of liver transaminase elevations under dalbavancin appears to be higher compared to vancomycin and linezolid and might be more prominent in patients with underlying liver disease^28,29^. Indeed, impaired right ventricular function negatively impacts liver function and was a likely cause for elevated GGT in two patients; potentially predisposing LVAD patients for GPT elevations. Thus, these results highlight the importance of liver function testing in prolonged dalbavancin courses^28,29^.

No other ADE were observed. Thrombocytopenia has rarely been reported under treatment of the structural similar teicoplanin, but no effect has been seen in this patient cohort^30^. Also, no influence on INR and dosing of the vitamin-k-antagonist phenprocoumon has been detected making dalbavancin a relatively safe alternative to other oral options^31–33^.

All patients included in this study had a persistent infection caused by *S. aureus* with one patient being treated for MRSA. As we did not observe breakthrough infections, we also did not observe resistance development in infection isolates. Clinical studies and interpretation criteria for antimicrobial susceptibility testing support the effectiveness of dalbavancin for infections caused by *Staphylococcus* spp. and *Streptococcus* spp.^21^. However, dalbavancin has been used to treat other Gram-positive organisms like *Corynebacterium* spp. and breakthrough infections occurred more frequently, as mentioned above^18^. Due to the limited number of reports, it remains unclear whether a potentially reduced susceptibility of other bacteria like *Corynebacterium* spp. or the different therapeutic strategies may be relevant in the context of breakthrough infections.

Despite the small sample size, overall more than 4000 days of dalbavancin therapy are analyzed. Currently, this report is one of the largest defined patient cohorts that received dalbavancin as long-term suppression therapy in VAD patients.

Due to the retrospective analysis in this register-based study, including a matched control group was not feasible in our setting. We therefore decided to include hospitalizations of each individual before and under dalbavancin therapy. This is clearly not the preferred study setting for the definite evaluation of dalbavancin in the long-term suppressive therapy of VAD infections. However, our findings may be regarded as exploratory and support the planning of larger prospective studies, ideally randomized controlled trials if possible, to determine the effects and outcomes of dalbavancin in this setting.

This analysis has several limitations. The sample size of 13 patients, all of them being treated for *S. aureus* infections in a single medical center, is too small to draw definite conclusions on clinical success or ADE prevalence under dalbavancin therapy. Likewise, the efficacy in infections caused by other Gram-positive bacteria cannot be judged. Furthermore, we conducted a retrospective analysis that is prone to a risk of bias e.g. selection bias. The use of dalbavancin in the presented setting is limited to patients with therapeutic failure under, or contraindications for oral antibiotic suppressive therapy, therefore being limited to a very specific clinical situation. In addition, this precondition and the retrospective nature of the study resulted in the absence of a matched comparator group as all patients fulfilling the criteria were treated with dalbavancin. To analyze the odds of hospitalization, we compared hospitalizations before and under dalbavancin therapy in the individual patients with a GLMM to account for the small sample size. With regard to this analysis, the statistical model assumes independence between each outcome which cannot be guaranteed due to stays in a hospital lasting several days. However, this is the preferred model choice for analysis on patient level compared to a paired t-test due to the small number of successes (hospital days) after the intervention (mostly 0). In addition, follow-up between patients was different because of different length of dalbavancin treatments. Therefore, results with regard to hospital utilization should be seen exploratory and need to be confirmed by additional studies. Thus, the results should be regarded as hypothesis generating and could be used for the design of larger, randomized trials involving more than one center, that are needed to definitely assess dalbavancin’s efficacy and safety for long-term suppression in VAD patients.

LVAD patients are prone to VAD-infections that are associated with recurrent hospitalizations and adverse outcomes. Taken together, in this exploratory study, the combination of initial surgical source control with intravenous antibiotic treatment followed by long-term suppressive therapy with repetitive cycles of dalbavancin significantly lowered the risk for hospitalization and no breakthrough infections occurred. Under long-term therapy, regular monitoring of liver function is warranted and no influence on INR or renal function has been detected. Given the small sample size, additional analyses in larger registers or, most preferable, randomized-controlled trials are needed to determine the best management strategy for this relevant clinical situation.

## Electronic supplementary material

Below is the link to the electronic supplementary material.

Supplementary Material 1

## Data Availability

The datasets used and analyzed during the current study are included in this published article and its supplementary information file.

## References

[1] Bock, M. et al. Attainment of target antibiotic levels by oral treatment of left-sided infective endocarditis: A POET substudy. *Clin. Infect. Dis.* **77**(2), 242–251. 10.1093/cid/ciad168 (2023). 36947131 10.1093/cid/ciad168

[2] Kuula, L. S. M., Viljemaa, K. M., Backman, J. T. & Blom, M. Fluoroquinolone-related adverse events resulting in health service use and costs: A systematic review. *PLoS ONE* **14**(4), e0216029. 10.1371/journal.pone.0216029 (2019). 31026286 10.1371/journal.pone.0216029 PMC6485715

[3] Fischer, H. D., Juurlink, D. N., Mamdani, M. M., Kopp, A. & Laupacis, A. Hemorrhage during warfarin therapy associated with cotrimoxazole and other urinary tract anti-infective agents: A population-based study. *Arch. Intern. Med.* **170**(7), 617–621. 10.1001/archinternmed.2010.37 (2010). 20386005 10.1001/archinternmed.2010.37

[4] Buffie, C. G. et al. Profound alterations of intestinal microbiota following a single dose of clindamycin results in sustained susceptibility to *Clostridium difficile*-induced colitis. *Infect. Immun.* **80**(1), 62–73. 10.1128/IAI.05496-11 (2012). 22006564 10.1128/IAI.05496-11 PMC3255689

[5] Zhang, J., Chen, L., Gomez-Simmonds, A., Yin, M. T. & Freedberg, D. E. Antibiotic-specific risk for community-acquired *Clostridioides difficile* infection in the United States from 2008 to 2020. *Antimicrob. Agents Chemother.* **66**(12), e0112922. 10.1128/aac.01129-22 (2022). 36377887 10.1128/aac.01129-22 PMC9764966

[6] Van Bambeke, F. Lipoglycopeptide antibacterial agents in gram-positive infections: A comparative review. *Drugs* **75**(18), 2073–2095. 10.1007/s40265-015-0505-8 (2015). 26603874 10.1007/s40265-015-0505-8

[7] Advanz Pharma. Summary of product characteristics. Xydalba 500 mg. Last accessed 26 Feb 2024.

[8] Senneville, E. et al. Expert opinion on dose regimen and therapeutic drug monitoring for long-term use of dalbavancin: Expert review panel. *Int. J. Antimicrob. Agents* **62**(5), 106960. 10.1016/j.ijantimicag.2023.106960 (2023). 37633424 10.1016/j.ijantimicag.2023.106960

[9] Rappo, U. et al. Dalbavancin for the treatment of osteomyelitis in adult patients: A randomized clinical trial of efficacy and safety. *Open Forum Infect. Dis.* **6**(1), ofy331. 10.1093/ofid/ofy331 (2019). 30648126 10.1093/ofid/ofy331 PMC6326511

[10] Hitzenbichler, F. et al. Dalbavancin as long-term suppressive therapy for patients with gram-positive bacteremia due to an intravascular source—A series of four cases. *Infection* **49**(1), 181–186. 10.1007/s15010-020-01526-0 (2021). 32965641 10.1007/s15010-020-01526-0 PMC7850995

[11] Rose, E. A. et al. Long-term use of a left ventricular assist device for end-stage heart failure. *N. Engl. J. Med.* **345**(20), 1435–1443. 10.1056/NEJMoa012175 (2001). 11794191 10.1056/NEJMoa012175

[12] Shah, P. et al. Twelfth interagency registry for mechanically assisted circulatory support report: Readmissions after left ventricular assist device. *Ann. Thorac. Surg.* **113**(3), 722–737. 10.1016/j.athoracsur.2021.12.011 (2022). 35007505 10.1016/j.athoracsur.2021.12.011 PMC8854346

[13] Zinoviev, R., Lippincott, C. K., Keller, S. C. & Gilotra, N. A. In full flow: Left ventricular assist device infections in the modern era. *Open Forum Infect. Dis.* **7**(5), ofaa124. 10.1093/ofid/ofaa124 (2020). 32405511 10.1093/ofid/ofaa124 PMC7209633

[14] Simeon, S. et al. Left ventricular assist device-related infections: A multicentric study. *Clin. Microbiol. Infect.* **23**(10), 748–751. 10.1016/j.cmi.2017.03.008 (2017). 28323195 10.1016/j.cmi.2017.03.008

[15] Kusne, S. et al. An ISHLT consensus document for prevention and management strategies for mechanical circulatory support infection. *J. Heart Lung Transplant.* **36**(10), 1137–1153. 10.1016/j.healun.2017.06.007 (2017). 28781010 10.1016/j.healun.2017.06.007

[16] Ekkelenkamp, M. B. et al. Therapy and outcome of *Staphylococcus aureus* infections of intracorporeal ventricular assist devices. *Artif. Organs* **42**(10), 983–991. 10.1111/aor.13159 (2018). 29675919 10.1111/aor.13159 PMC6220828

[17] Mansoor, A. E., Krekel, T. & Cabrera, N. L. Experience with dalbavancin for long-term antimicrobial suppression of left ventricular assist device infections. *Transpl. Infect. Dis.* **25**(4), e14068. 10.1111/tid.14068 (2023). 37159539 10.1111/tid.14068

[18] Rowe, S., Green, S., Albrecht, B. & Pouch, S. M. Long-term dalbavancin for suppression of gram-positive chronic left ventricular assist device infections. *Open Forum Infect. Dis.* **10**(11), ofad537. 10.1093/ofid/ofad537 (2023). 38023541 10.1093/ofid/ofad537 PMC10673638

[19] von Elm, E. et al. Strengthening the reporting of observational studies in epidemiology (STROBE) statement: Guidelines for reporting observational studies. *BMJ* **335**(7624), 806–808. 10.1136/bmj.39335.541782.AD (2007). 17947786 10.1136/bmj.39335.541782.AD PMC2034723

[20] Chiriac, U. et al. Validation and application of an HPLC-UV method for routine therapeutic drug monitoring of dalbavancin. *Antibiotics (Basel)* **11**(5), 541. 10.3390/antibiotics11050541 (2022). 35625185 10.3390/antibiotics11050541 PMC9137512

[21] The European Committee on Antimicrobial Susceptibility Testing. Breakpoint tables for interpretation of MICs and zone diameters. Version 14.0, 2024. http://www.eucast.org.

[22] Bernhardt, A. M. et al. Prevention and early treatment of driveline infections in ventricular assist device patients—The DESTINE staging proposal and the first standard of care protocol. *J. Crit. Care* **56**, 106–112. 10.1016/j.jcrc.2019.12.014 (2020). 31896443 10.1016/j.jcrc.2019.12.014

[23] Kidney Disease Improving Global Outcomes (KDIGO). Clinical practice guideline for the evaluation and management of chronic kidney disease. *Kidney Int.* **3**(1), 5–14 (2012).

[24] Chiriac, U. et al. Model-based dose identification of dalbavancin for long-term suppressive outpatient treatment of ventricular assist device infections. *Antibiotics (Basel).* **13**(11), 1103. 10.3390/antibiotics13111103 (2024). 39596796 10.3390/antibiotics13111103 PMC11591545

[25] Pallotto, C., Tordi, S., Pantanella, R., Rosignoli, D. & Francisci, D. Dalbavancin as chronic antibiotic suppression therapy for left ventricular assist device driveline infection due to methicillin-resistant *Staphylococcus aureus*: A case report. *J. Chemother.* **35**(5), 465–469. 10.1080/1120009X.2022.2136426 (2023). 36281721 10.1080/1120009X.2022.2136426

[26] Hervochon, C. et al. Dalbavancin plasma concentrations in 133 patients: A PK/PD observational study. *J. Antimicrob. Chemother.* **78**(12), 2919–2925. 10.1093/jac/dkad331 (2023). 37864551 10.1093/jac/dkad331

[27] Turner, N. A. et al. Dalbavancin as an option for treatment of *S. aureus* bacteremia (DOTS): Study protocol for a phase 2b, multicenter, randomized, open-label clinical trial. *Trials* **23**(1), 407. 10.1186/s13063-022-06370-1 (2022). 35578360 10.1186/s13063-022-06370-1 PMC9109297

[28] Simonetti, O., Rizzetto, G., Molinelli, E., Cirioni, O. & Offidani, A. Review: A safety profile of dalbavancin for on- and off-label utilization. *Ther. Clin. Risk Manag.* **17**, 223–232. 10.2147/TCRM.S271445 (2021). 33790563 10.2147/TCRM.S271445 PMC7997409

[29] FDA Advisory committee on anti-infective drugs. Introductory Remarks—Anti-Infective Drugs Advisory Committee Meeting for Dalbavancin. March 31st 2014. NDA 21–883: Dalbavancin for Injection. From: http://web.archive.org/web/20161024125436/http://www.fda.gov/downloads/AdvisoryCommittees/CommitteesMeetingMaterials/Drugs/Anti-InfectiveDrugsAdvisoryCommittee/UCM392559.pdf. Last accessed 26 Feb 2024.

[30] Elajez, R. et al. Thrombocytopenia associated with teicoplanin use: A retrospective observational study. *Ann. Pharmacother.*10.1177/10600280221078123 (2022). 35179076 10.1177/10600280221078123

[31] Ahmed, A., Stephens, J. C., Kaus, C. A. & Fay, W. P. Impact of preemptive warfarin dose reduction on anticoagulation after initiation of trimethoprim-sulfamethoxazole or levofloxacin. *J. Thromb. Thrombolysis* **26**(1), 44–48. 10.1007/s11239-007-0164-z (2008). 17985084 10.1007/s11239-007-0164-z

[32] Penning-van Beest, F. J., Koerselman, J. & Herings, R. M. Risk of major bleeding during concomitant use of antibiotic drugs and coumarin anticoagulants. *J. Thromb. Haemost.* **6**(2), 284–290. 10.1111/j.1538-7836.2008.02844.x (2008). 18031295 10.1111/j.1538-7836.2008.02844.x

[33] Schelleman, H. et al. Warfarin with fluoroquinolones, sulfonamides, or azole antifungals: Interactions and the risk of hospitalization for gastrointestinal bleeding. *Clin. Pharmacol. Ther.* **84**(5), 581–588. 10.1038/clpt.2008.150 (2008). 18685566 10.1038/clpt.2008.150 PMC2574587

