# Supplementary material for: Exploratory analysis of long-term suppressive therapy with dalbavancin in ventricular assist device infections caused by Staphylococcus aureus
Source: Sci Rep. 2025 May 3;15:15515. doi: 10.1038/s41598-025-99112-7 (PMC12049434; doi:10.1038/s41598-025-99112-7)
Supplement: Supplementary file 1 — Supplementary Material 1 [file 41598_2025_99112_MOESM1_ESM.pdf]

Supplementary Information

Exploratory analysis of long-term suppressive therapy with dalbavancin in ventricular assist device infections caused by *Staphylococcus aureus*

Benedict Morath[1#], Sabrina Klein[2,3#*], Ute Chiriac[1], Yvonne Müller[4], Lisa Koeppel[5], Otto Frey[6], Heike Lanzinger[6], Philipp Schlegel[7], Sonja Hamed[7], Dennis Nurjadi[2,3,8, 9], Philipp Ehlermann[7], Matthias Karck[4], Anna L. Meyer[4]

[#]both authors contributed equally

*corresponding author

Table of contents

Figures 3

Figure SI1: Wound score and wound state. 3

Figure SI2: Internalized normalized ratio of patients under dalbavancin therapy. 4

C-reactive protein, leucocytes, GOT, GPT, GGT, wound score and wound state of the individual patients 5

Patient A 5

Patient B 7

Patient C 9

Patient D 11

Patient E 13

Patient F 15

Patient G 17

Patient H 19

Patient I 21

Patient J 23

Patient K 25

Patient L 27

Patient M 29

Tables 31
Table SI1: Diagnoses and comorbidities of the included patients. 31
Table SI2: Individual cumulative hospitalization days of all patients before and under dalbavancin 33
Table SI3: Microbiological results of the included patients under dalbavancin therapy. 34
Renal function of the individual patients 36
Patient A 36
Patient B 36
Patient C 37
Patient D 37
Patient E 38
Patient F 39
Patient G 39
Patient H 40
Patient I 41
Patient J 42
Patient K 42
Patient L 43
Patient M 43

Figures

Figure SI1: Wound score and wound state.

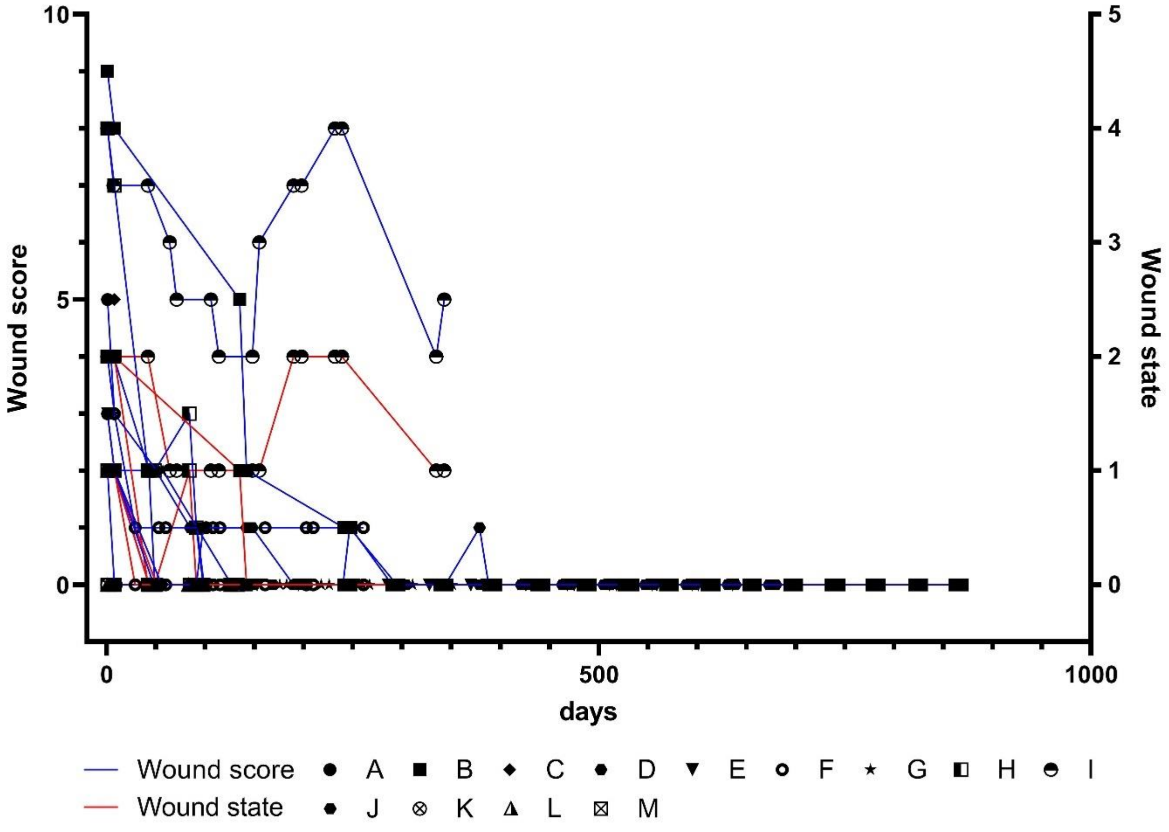

Fig. SI1: Wound score and wound state according to the adjusted DESTINE-criteria

Figure SI2: Internalized normalized ratio of patients under dalbavancin therapy.

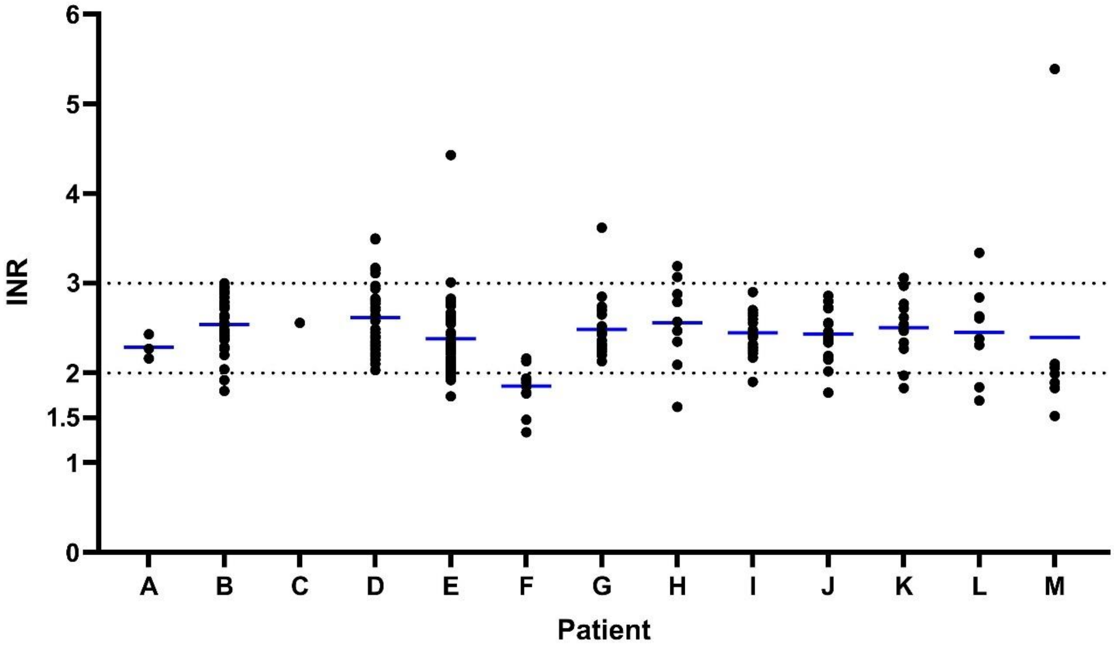

Fig. SI2: Internationalized normalized ratio (INR) of patients under dalbavancin long-term therapy. Patient F had a target of 1.7-2.0 due to gastro-intestinal bleeding.

C-reactive protein, leucocytes, GOT, GPT, GGT, wound score and wound state of the individual patients

Patient A

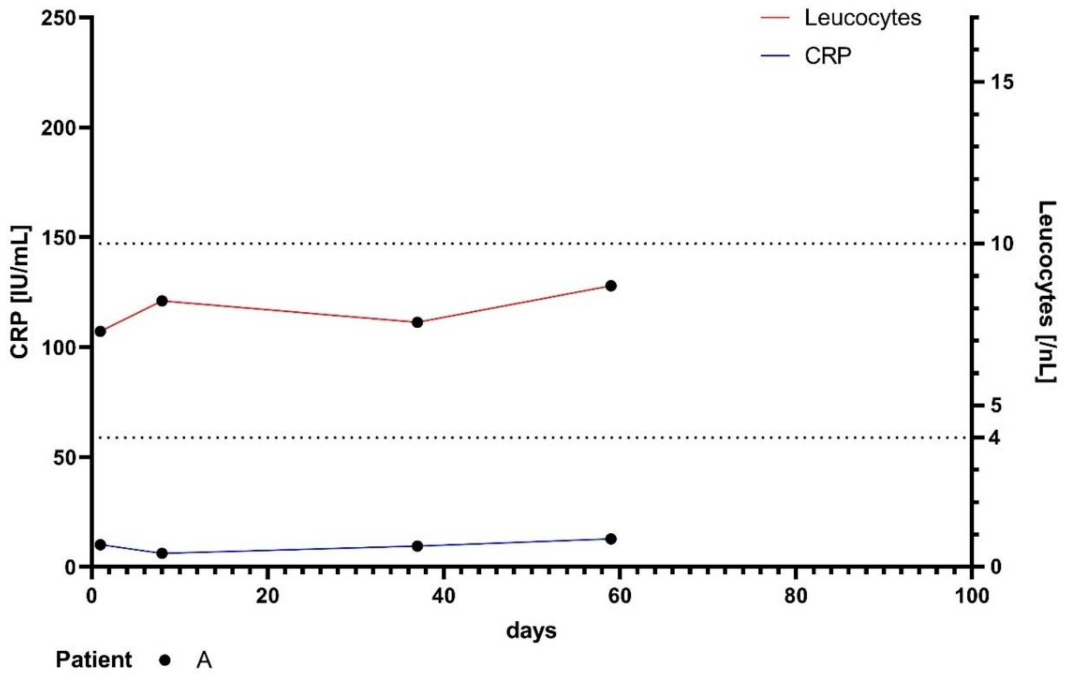

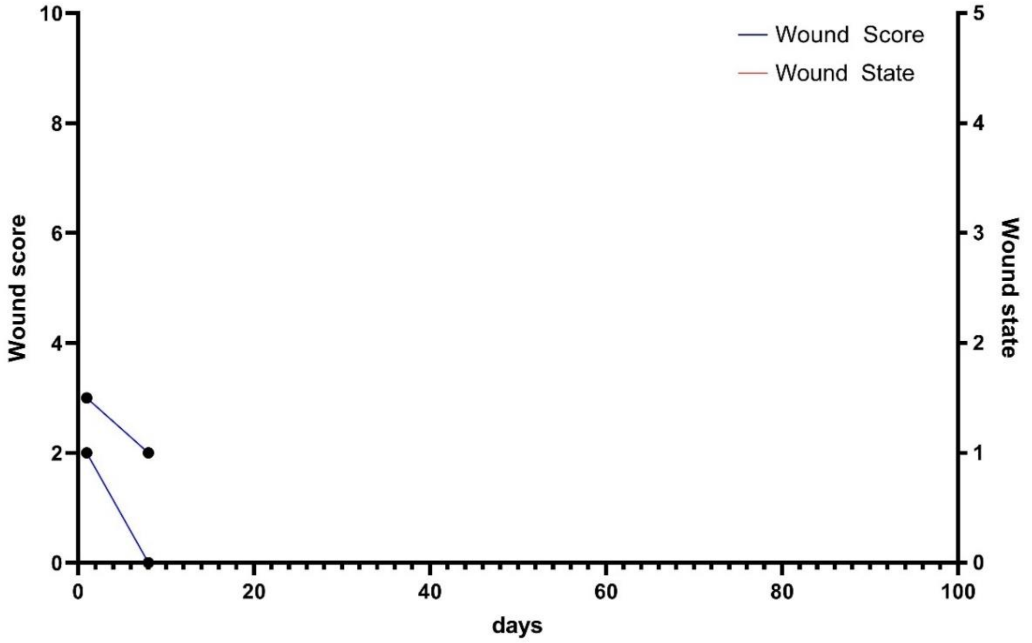

Fig. SI3: C-reactive protein, leucocytes and wound score and wound state according to the adjusted DESTINE-criteria of Patient A . **CRP:** C-reactive protein

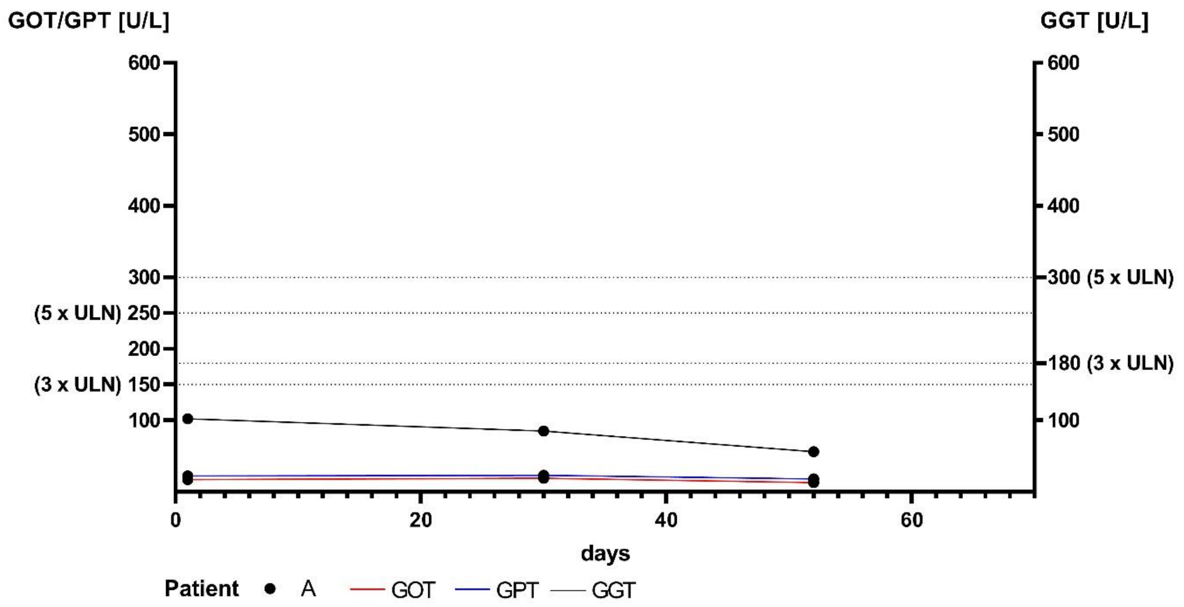

Fig.SI4: GOT, GPT, and GGT of patient A.
GOT: Glutamate-oxalacetate transferase | **GPT:** Glutamate-pyruvate transferase| **GGT:** Gamma-glutamine transferase | **ULN:** Upper limit of normal |

Patient B

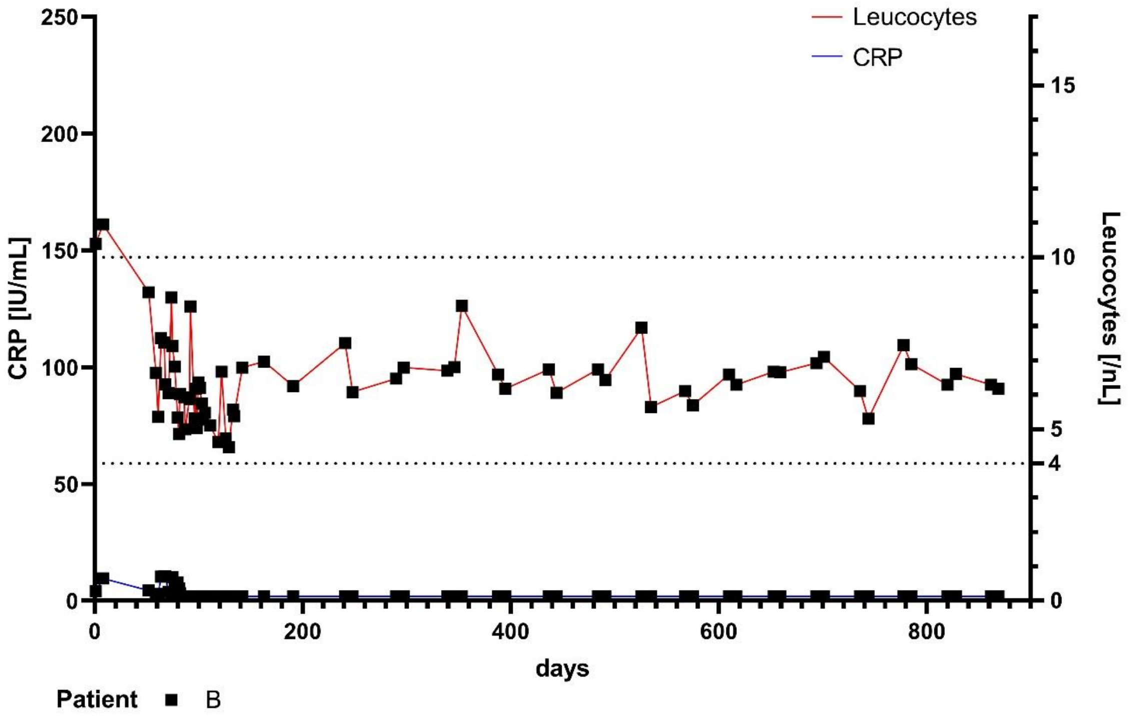

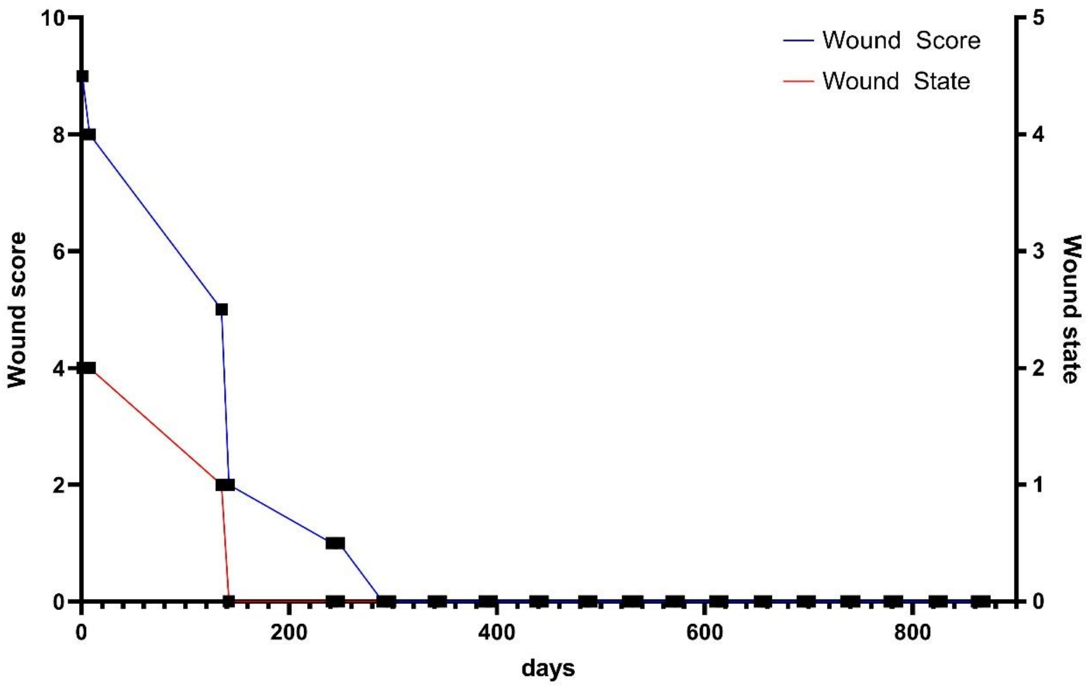

Fig. SI5: C-reactive protein, leucocytes and wound score and wound state according to the adjusted DESTINE-criteria of Patient B. **CRP:** C-reactive protein

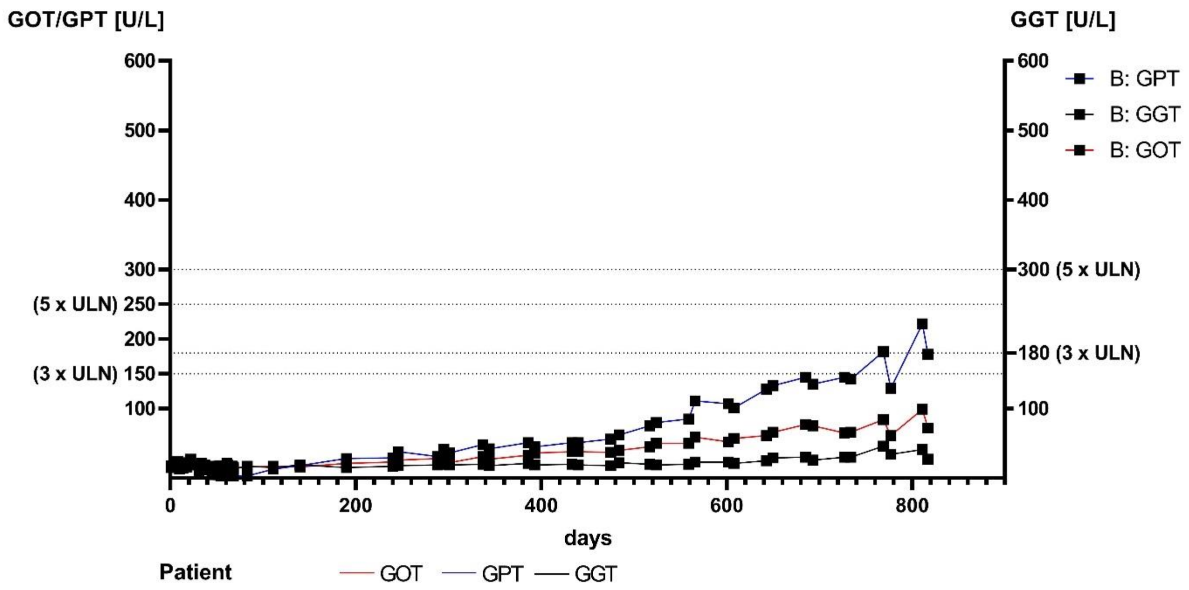

Fig. SI6: GOT, GPT, and GGT of patient B.

GOT: Glutamate-oxalacetate transferase | **GPT:** Glutamate-pyruvate transferase| **GGT:** Gamma-glutamine transferase | **ULN:** Upper limit of normal |

Patient C

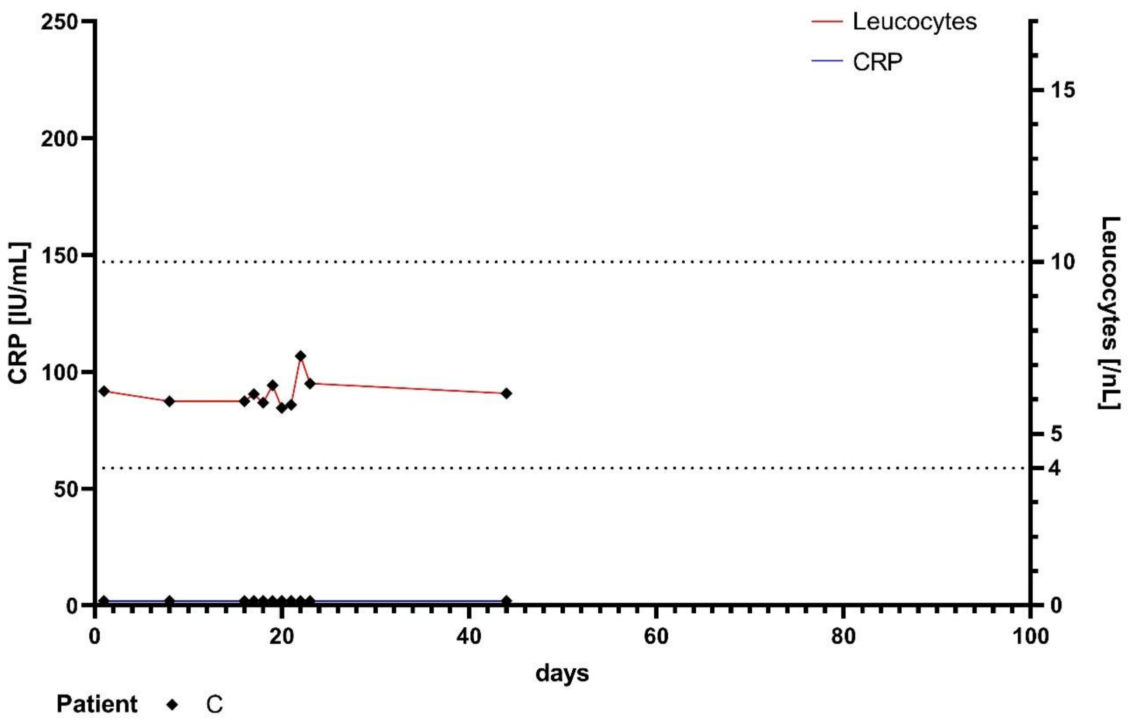

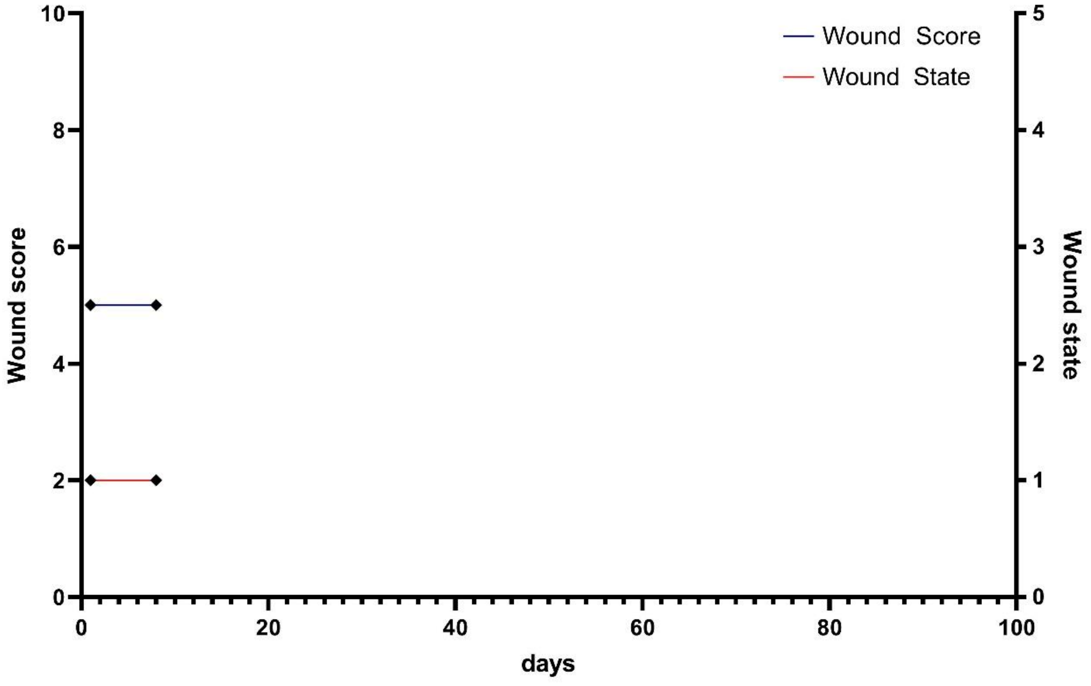

Fig.SI7: C-reactive protein, leucocytes and wound score and wound state according to the adjusted DESTINE-criteria of Patient C. **CRP:** C-reactive protein

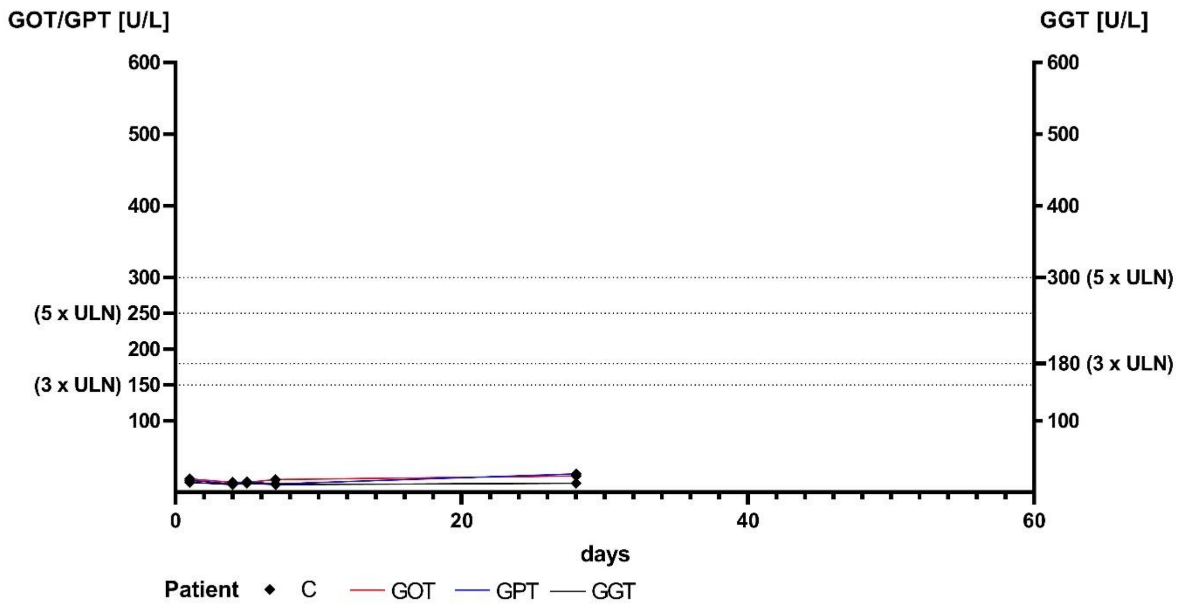

Fig. SI6: GOT, GPT, and GGT of patient C.
GOT: Glutamate-oxalacetate transferase | **GPT:** Glutamate-pyruvate transferase| **GGT:** Gamma-glutamine transferase | **ULN:** Upper limit of normal |

Patient D

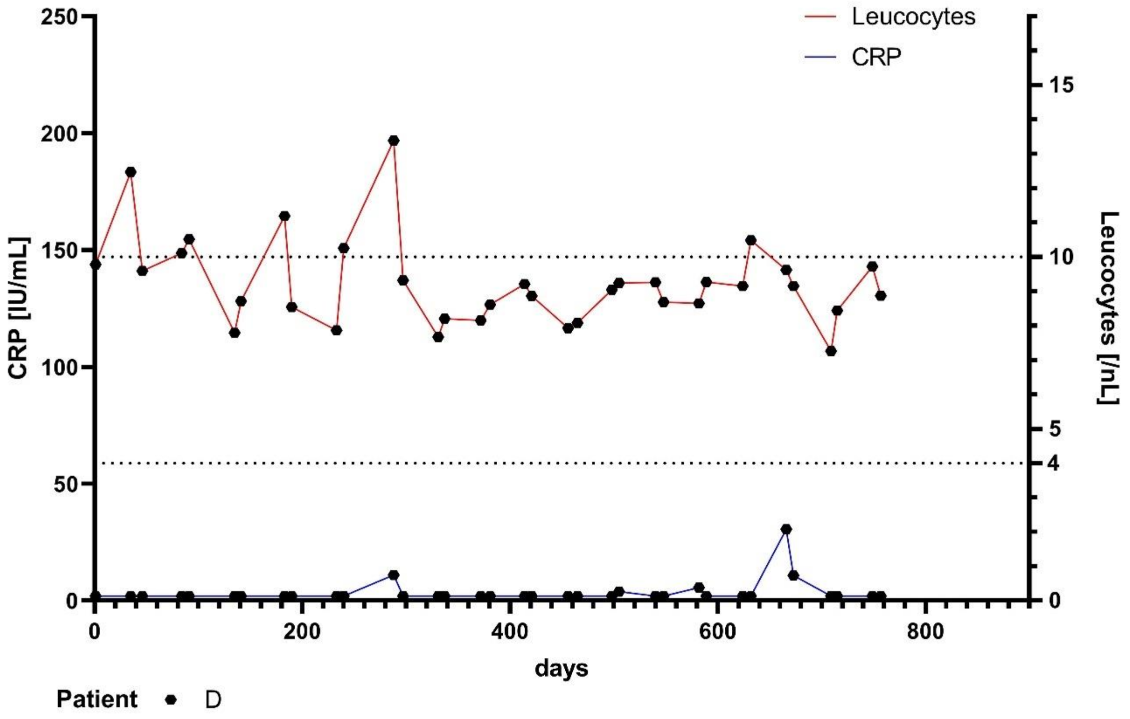

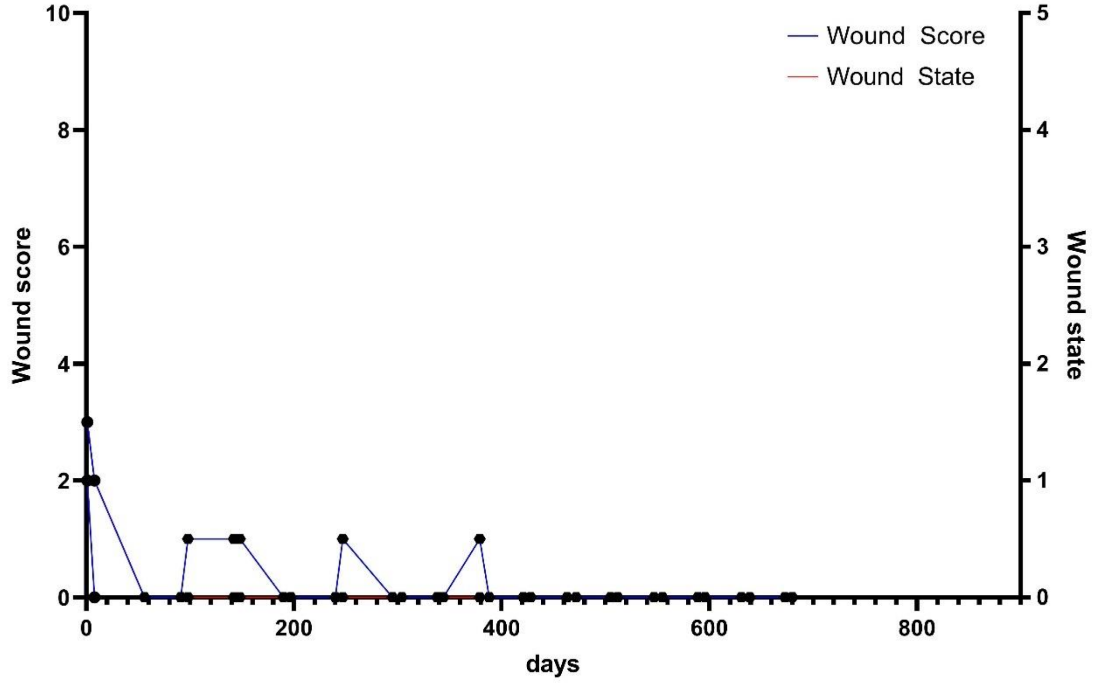

Fig.SI7: C-reactive protein, leucocytes and wound score and wound state according to the adjusted DESTINE-criteria of Patient D. **CRP:** C-reactive protein

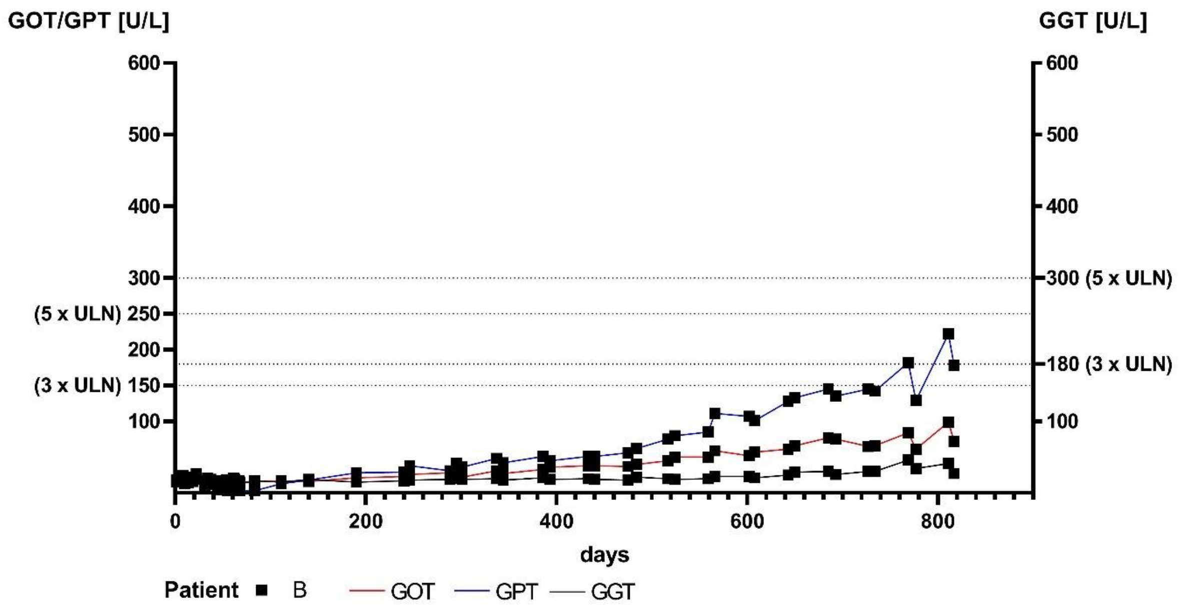

Fig. SI8: GOT, GPT, and GGT of patient D.
GOT: Glutamate-oxalacetate transferase | **GPT:** Glutamate-pyruvate transferase| **GGT:** Gamma-glutamine transferase | **ULN:** Upper limit of normal |

Patient E

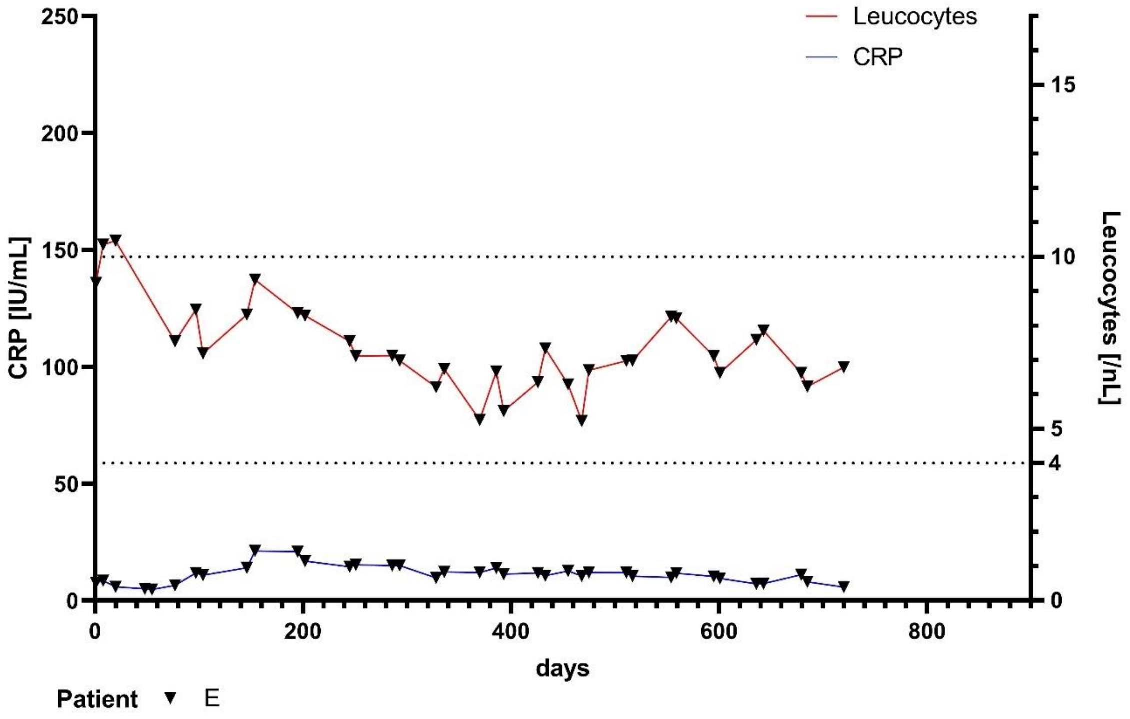

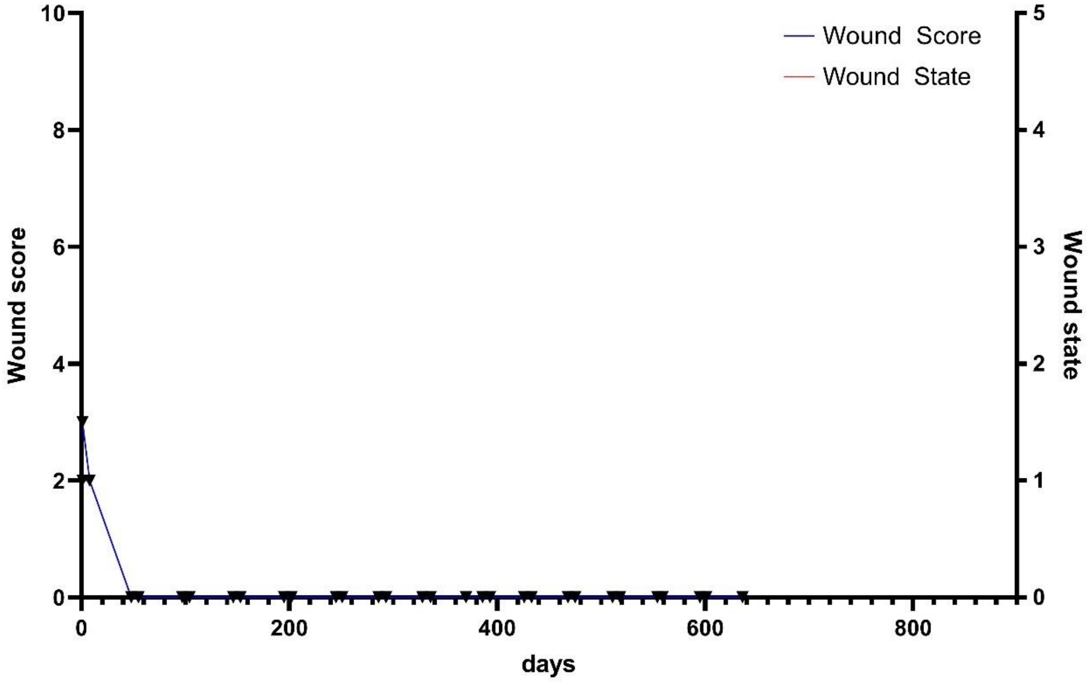

Fig. SI9: C-reactive protein, leucocytes and wound score and wound state according to the adjusted DESTINE-criteria of Patient E. **CRP:** C-reactive protein

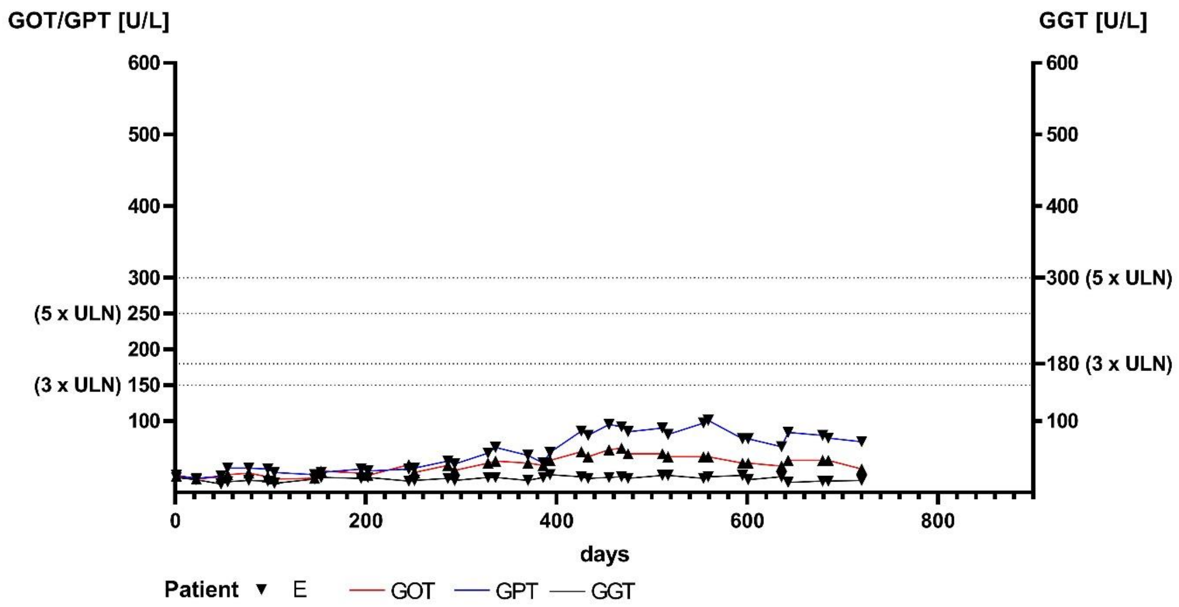

Fig. SI10: GOT, GPT, and GGT of patient E.

GOT: Glutamate-oxalacetate transferase | **GPT:** Glutamate-pyruvate transferase| **GGT:** Gamma-glutamine transferase | **ULN:** Upper limit of normal |

Patient F

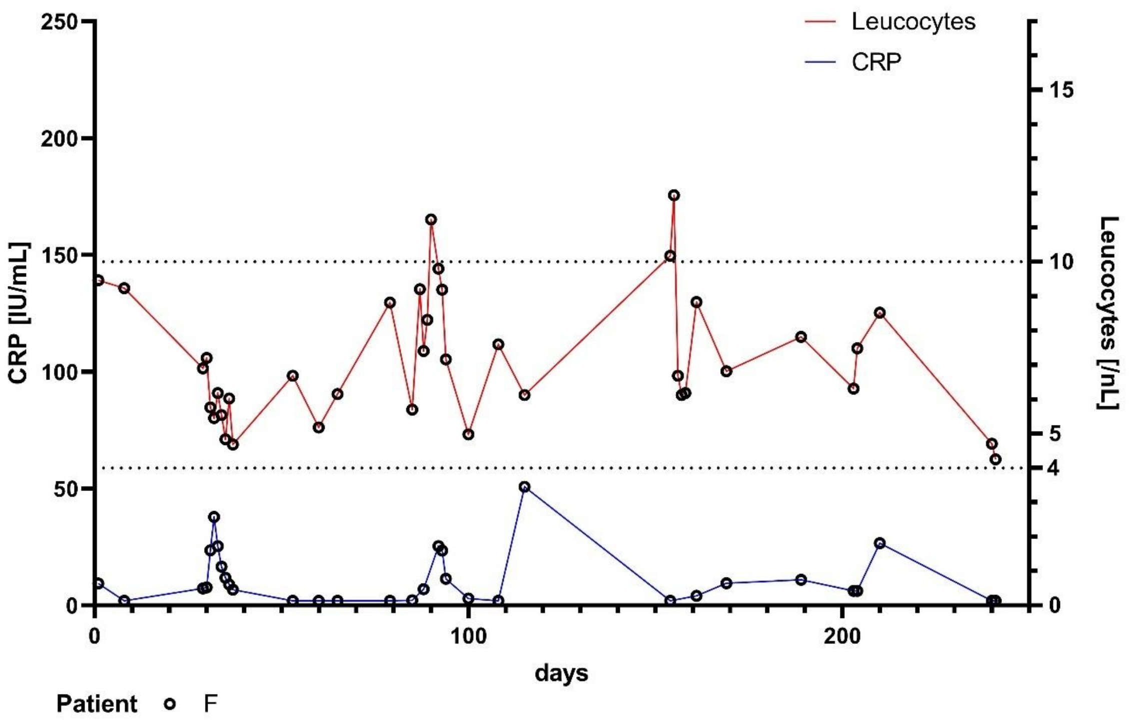

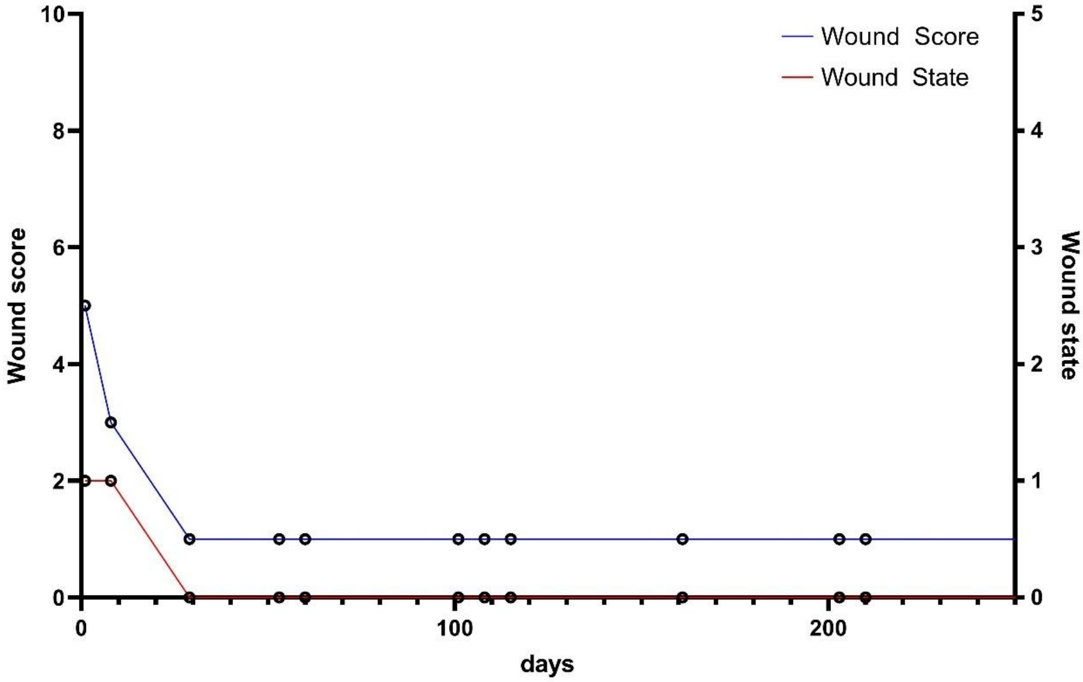

Fig. SI11: C-reactive protein, leucocytes and wound score and wound state according to the adjusted DESTINE-criteria of Patient F. **CRP:** C-reactive protein

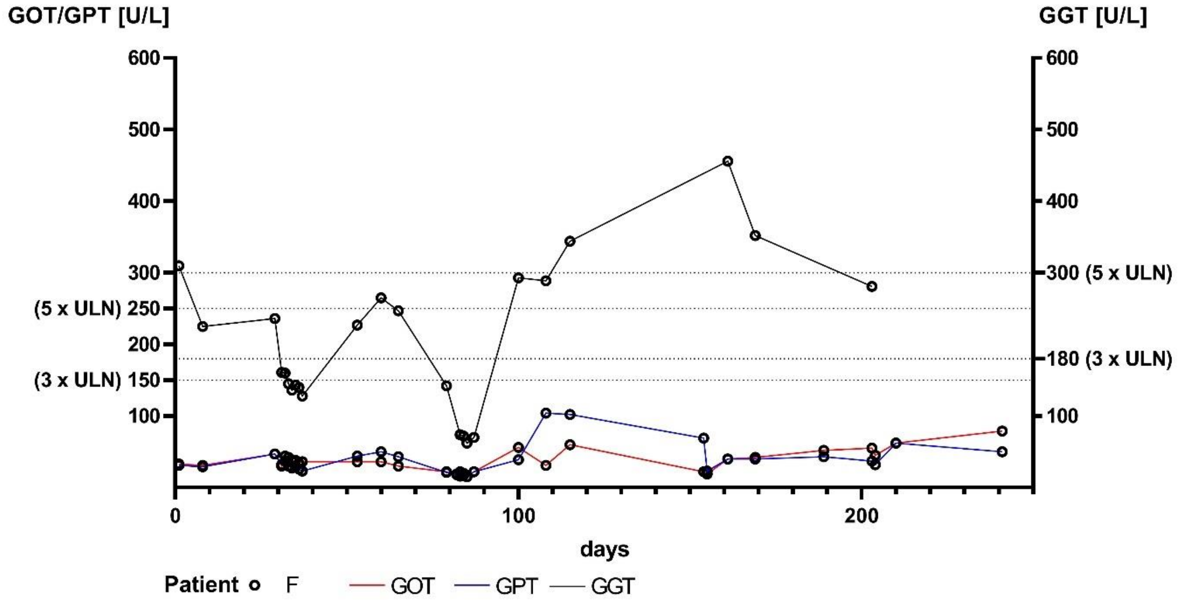

Fig. SI12: GOT, GPT, and GGT of patient F.
GOT: Glutamate-oxalacetate transferase | **GPT:** Glutamate-pyruvate transferase| **GGT:** Gamma-glutamine transferase | **ULN:** Upper limit of normal |

Patient G

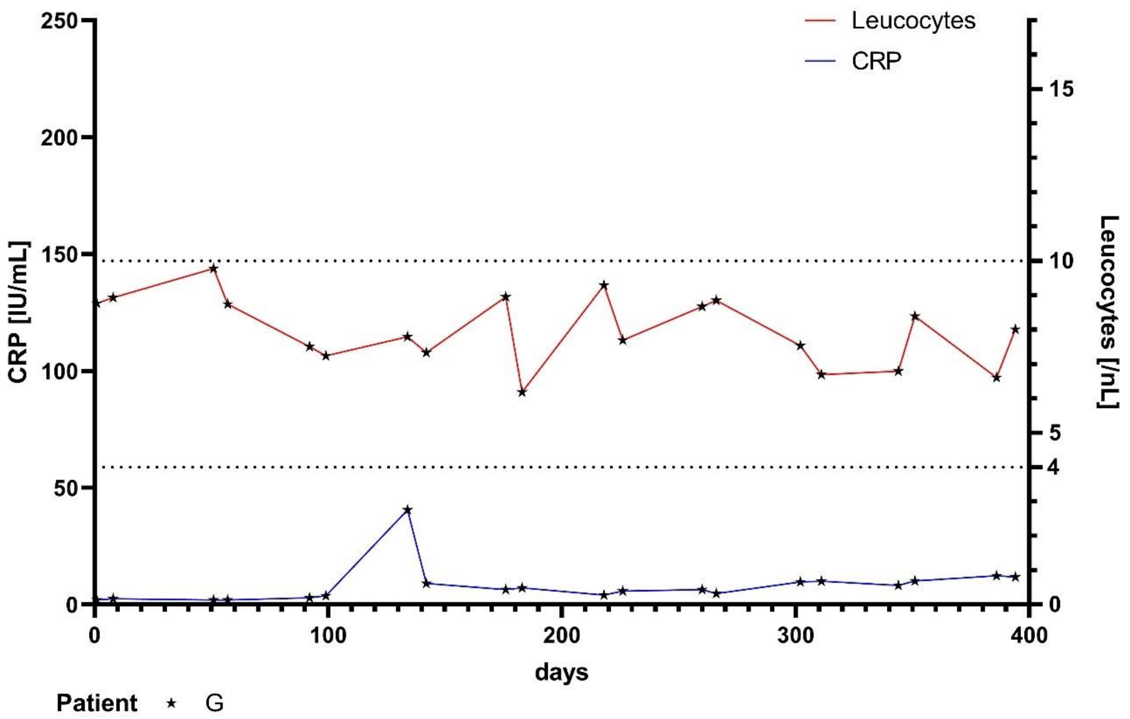

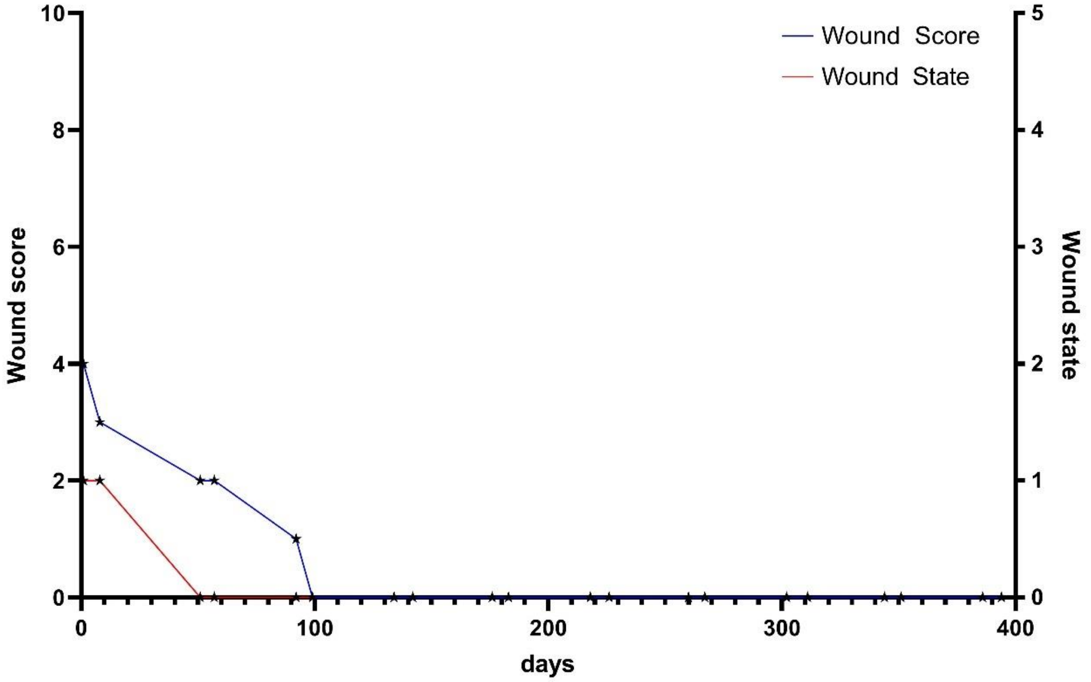

Fig. SI13: C-reactive protein, leucocytes and wound score and wound state according to the adjusted DESTINE-criteria of Patient G. **CRP:** C-reactive protein

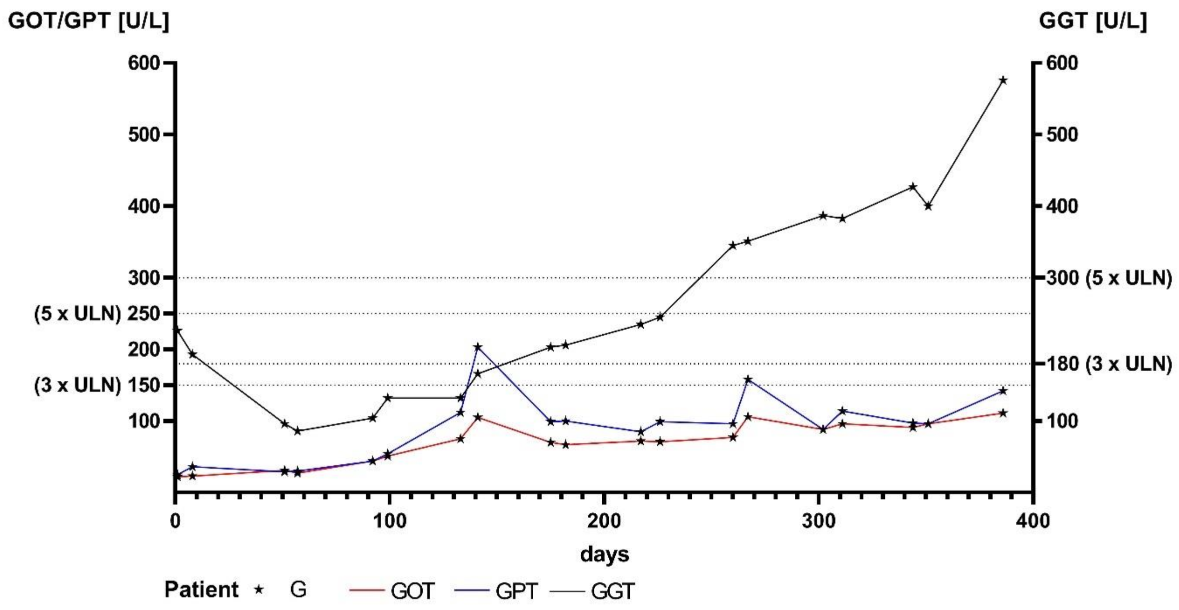

Fig. SI14: GOT, GPT, and GGT of patient G.
GOT: Glutamate-oxalacetate transferase | **GPT:** Glutamate-pyruvate transferase| **GGT:** Gamma-glutamine transferase | **ULN:** Upper limit of normal |

Patient H

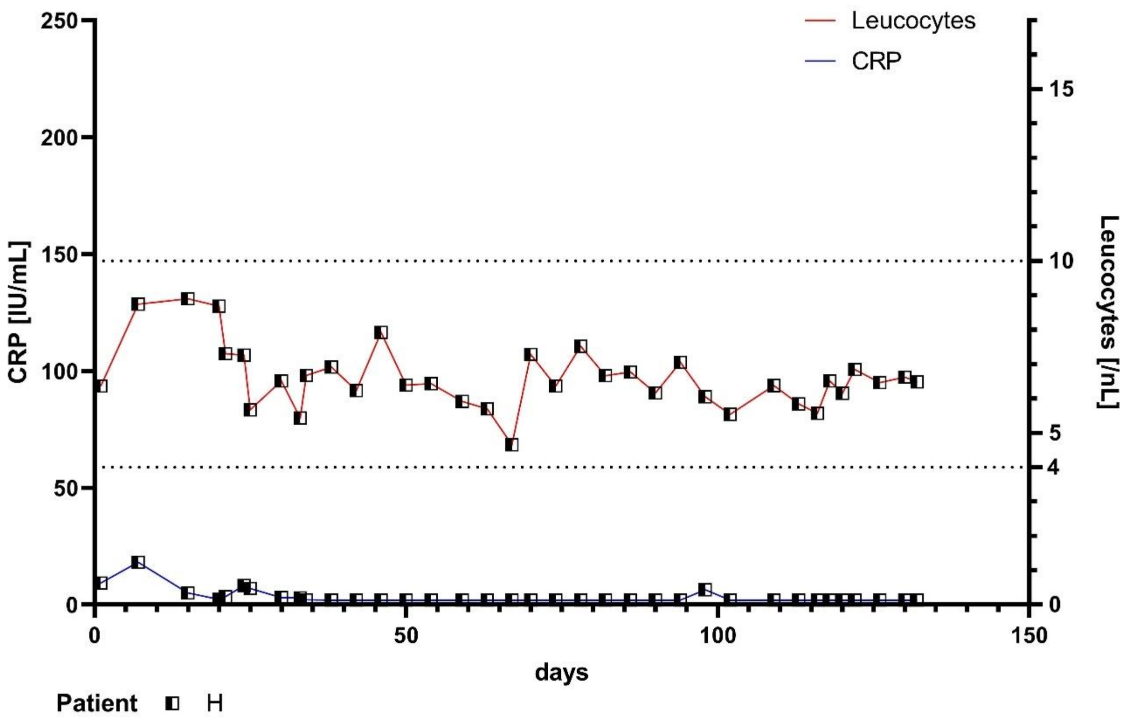

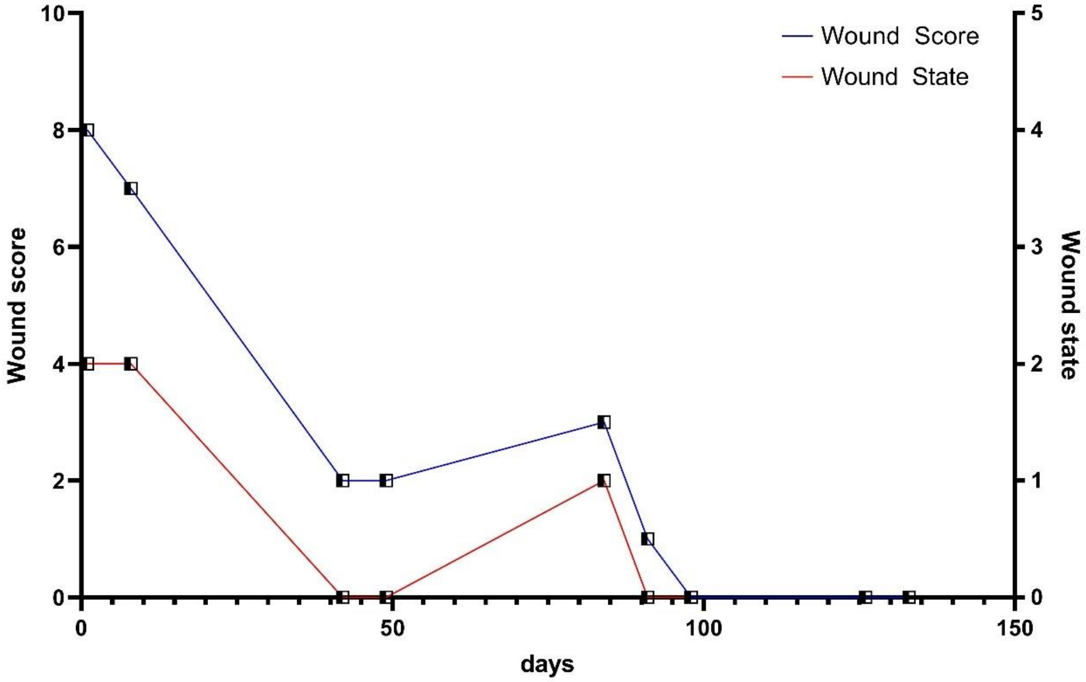

Fig. SI15: C-reactive protein, leucocytes and wound score and wound state according to the adjusted DESTINE-criteria of Patient H. **CRP:** C-reactive protein

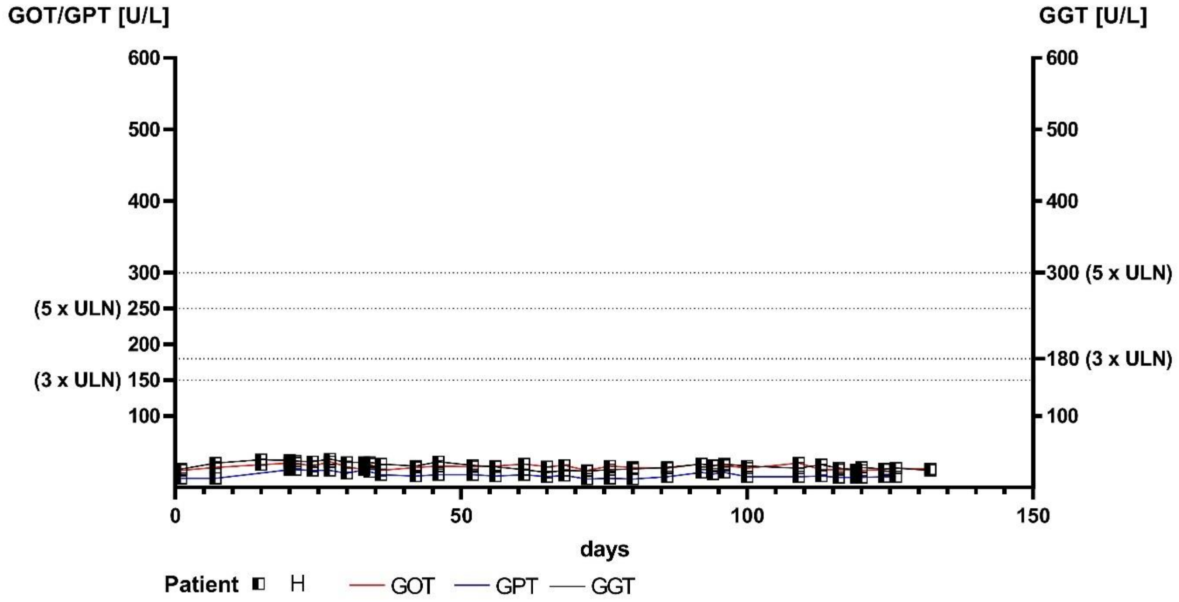

Fig. SI16: GOT, GPT, and GGT of patient H.
GOT: Glutamate-oxalacetate transferase | **GPT:** Glutamate-pyruvate transferase| **GGT:** Gamma-glutamine transferase | **ULN:** Upper limit of normal |

Patient I

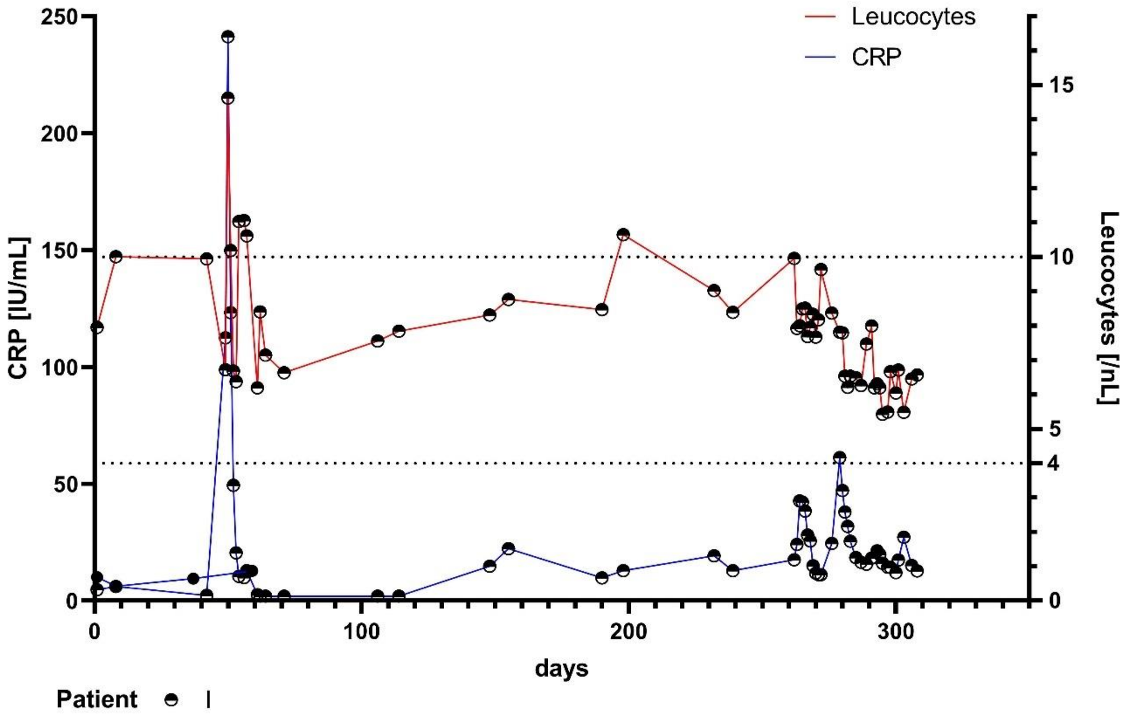

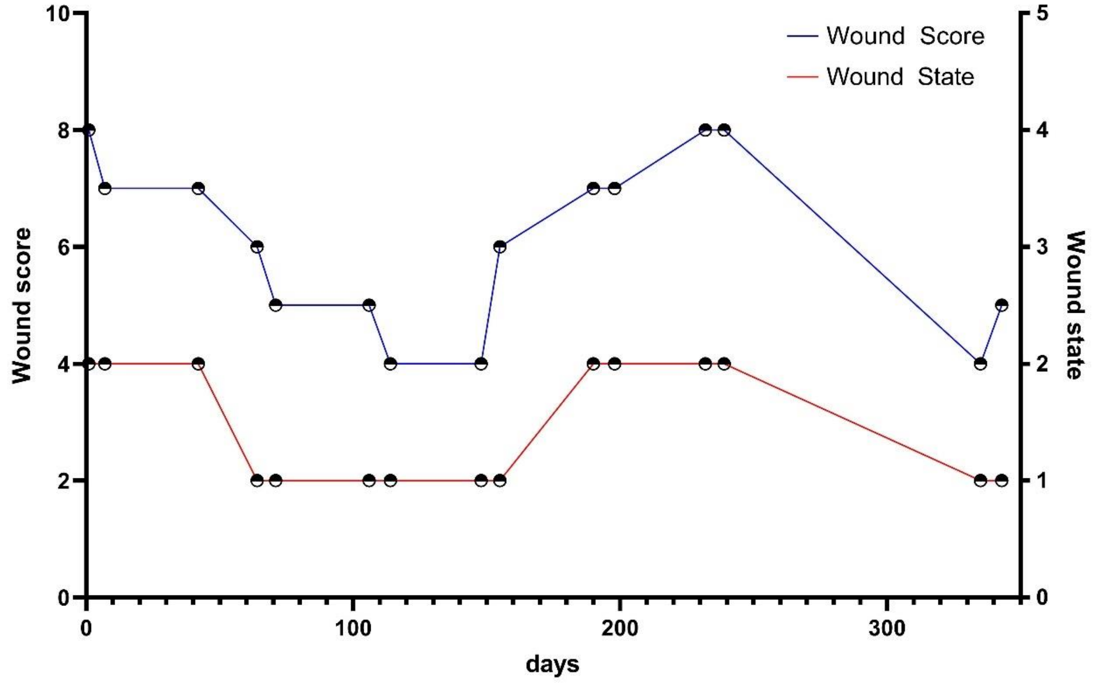

Fig. SI17: C-reactive protein, leucocytes and wound score and wound state according to the adjusted DESTINE-criteria of Patient I. **CRP:** C-reactive protein

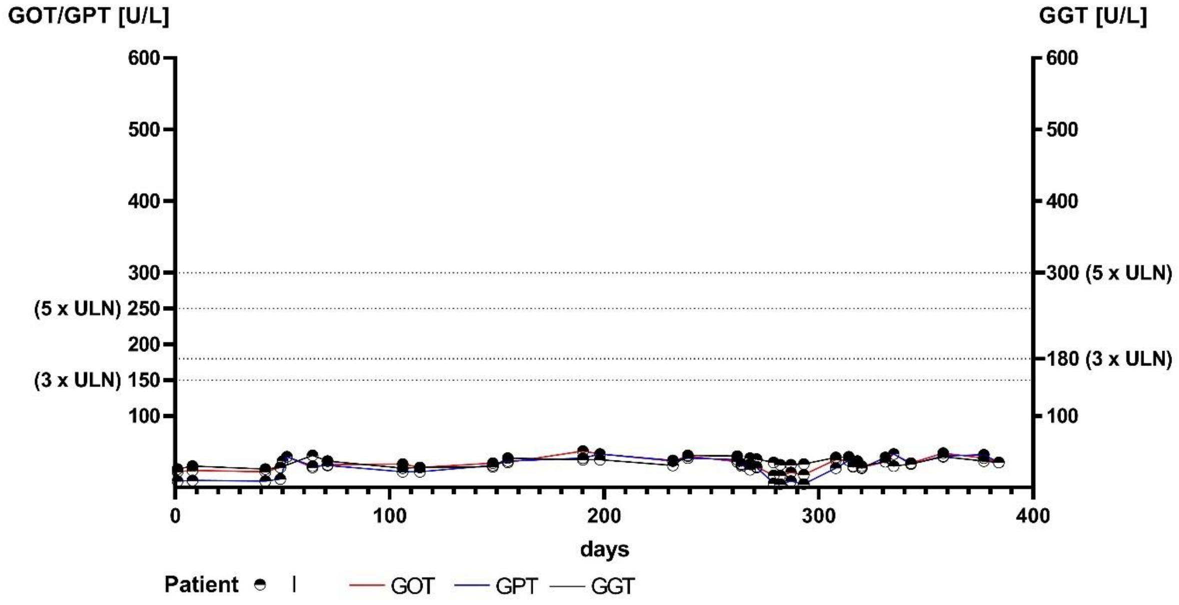

Fig. SI18: GOT, GPT, and GGT of patient I.
GOT: Glutamate-oxalacetate transferase | **GPT:** Glutamate-pyruvate transferase| **GGT:** Gamma-glutamine transferase | **ULN:** Upper limit of normal |

Patient J

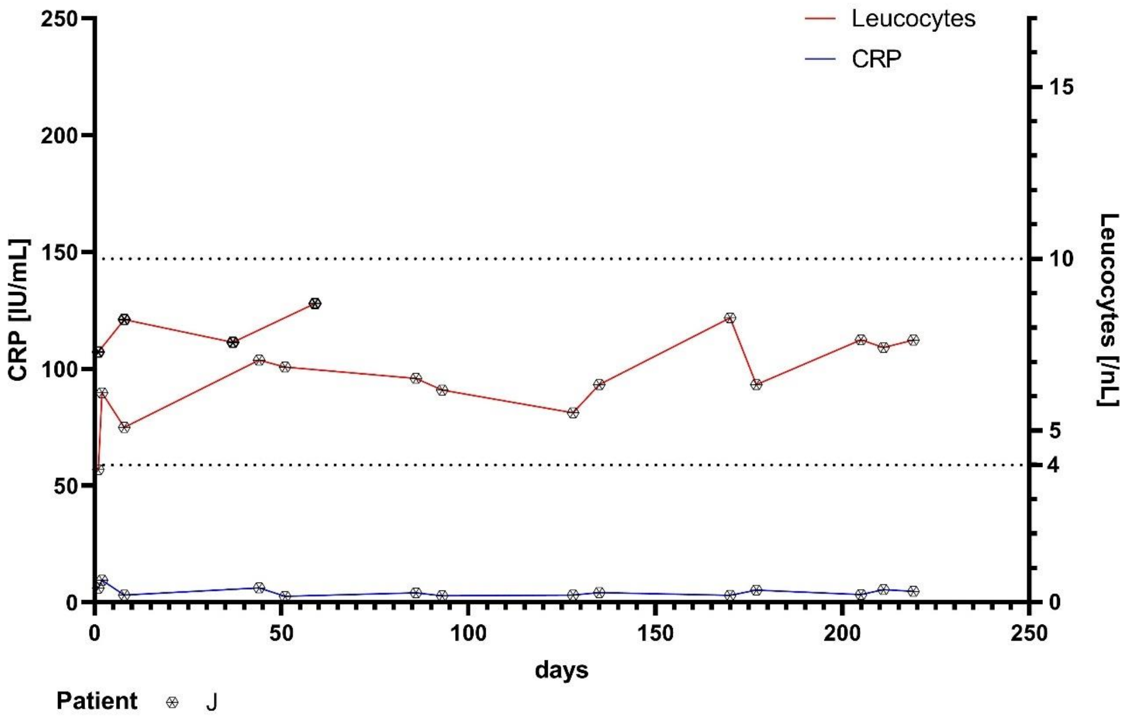

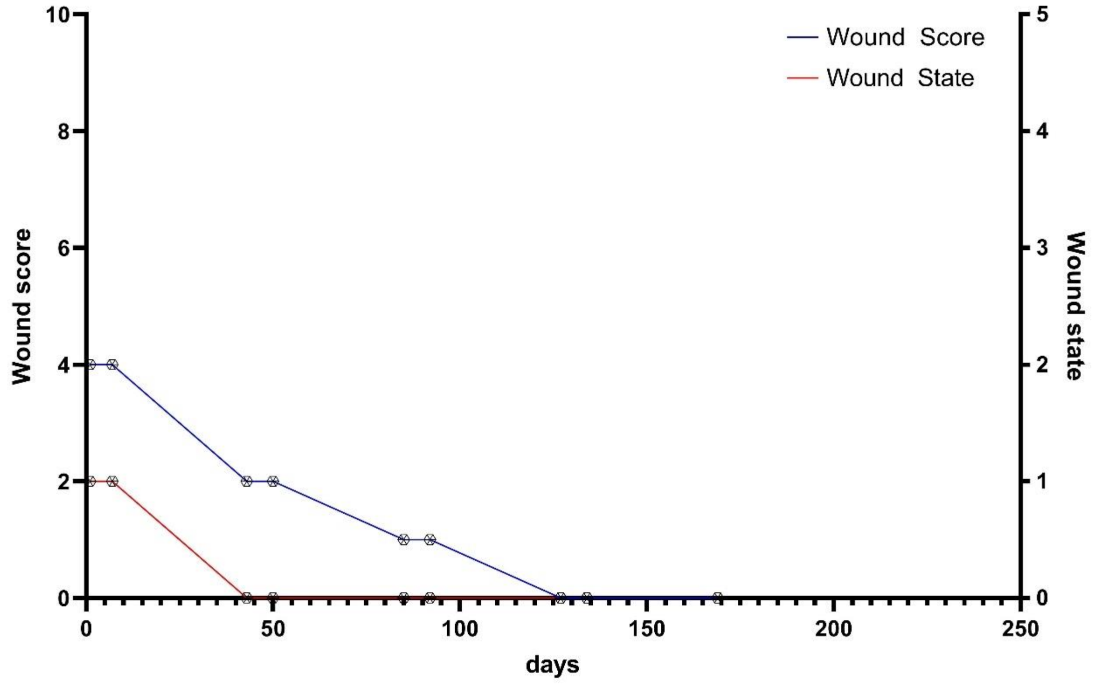

Fig. SI19: C-reactive protein, leucocytes and wound score and wound state according to the adjusted DESTINE-criteria of Patient J. **CRP:** C-reactive protein

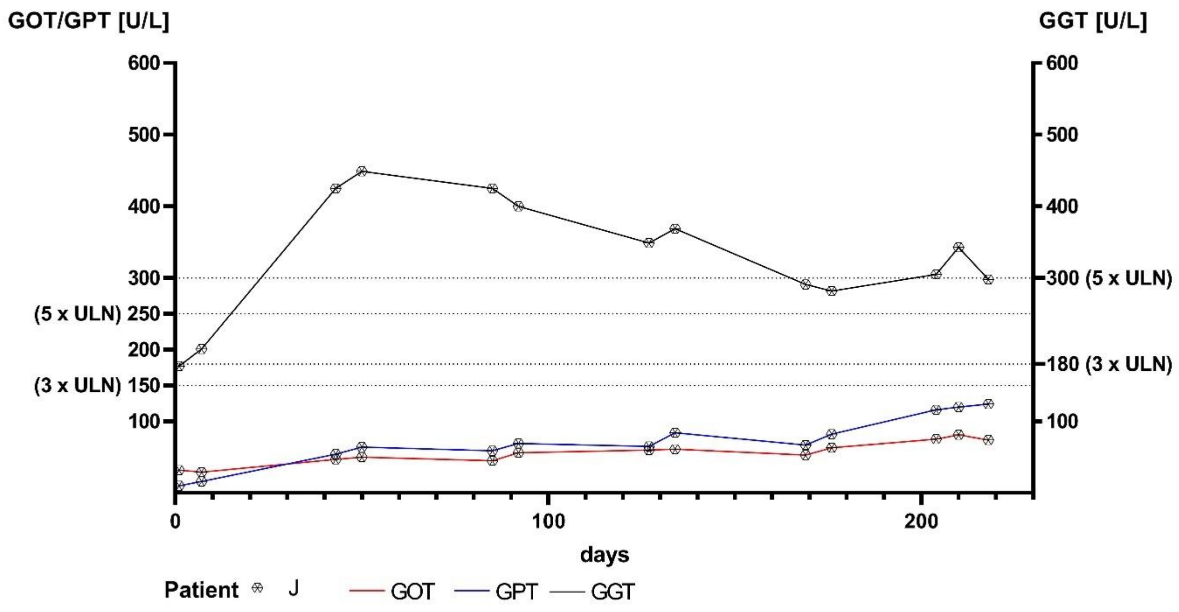

Fig. SI20: GOT, GPT, and GGT of patient J.
GOT: Glutamate-oxalacetate transferase | **GPT:** Glutamate-pyruvate transferase| **GGT:** Gamma-glutamine transferase | **ULN:** Upper limit of normal |

Patient K

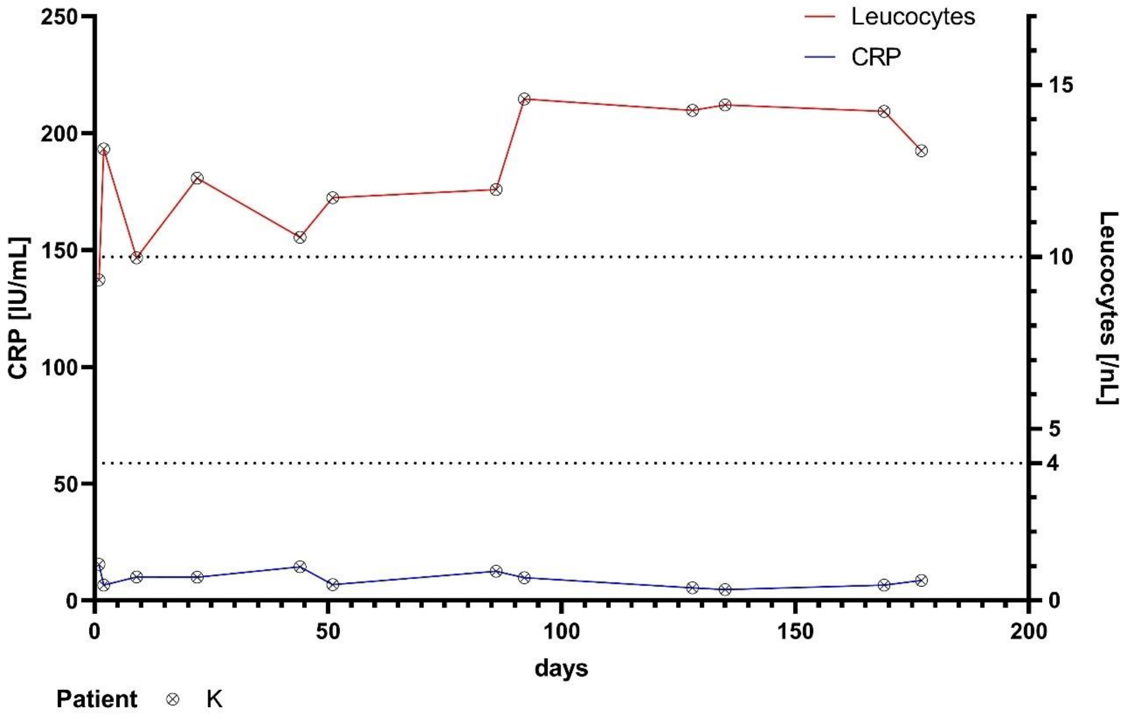

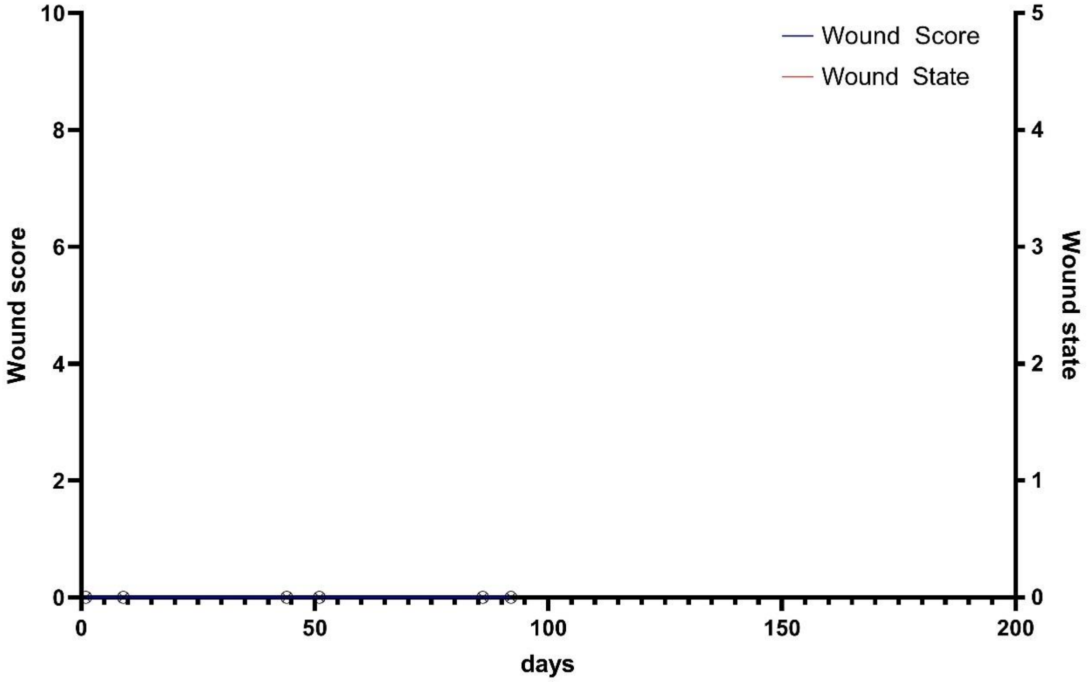

Fig. SI21: C-reactive protein, leucocytes and wound score and wound state according to the adjusted DESTINE-criteria of Patient K. **CRP:** C-reactive protein

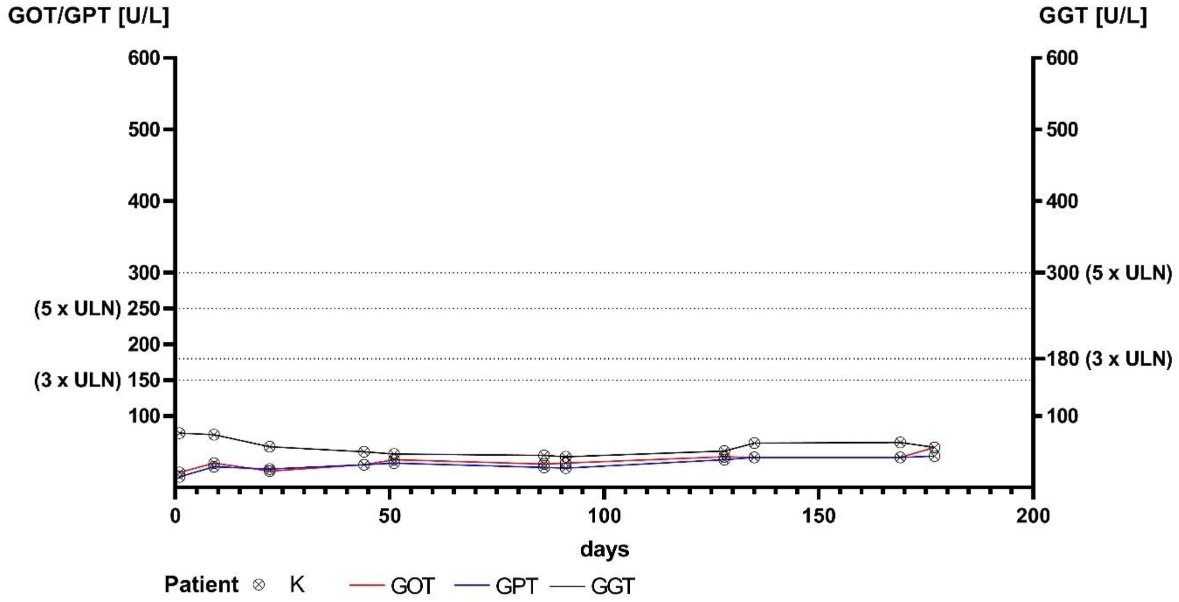

Fig. SI22: GOT, GPT, and GGT of patient K.
GOT: Glutamate-oxalacetate transferase | **GPT:** Glutamate-pyruvate transferase| **GGT:** Gamma-glutamine transferase | **ULN:** Upper limit of normal |

Patient L

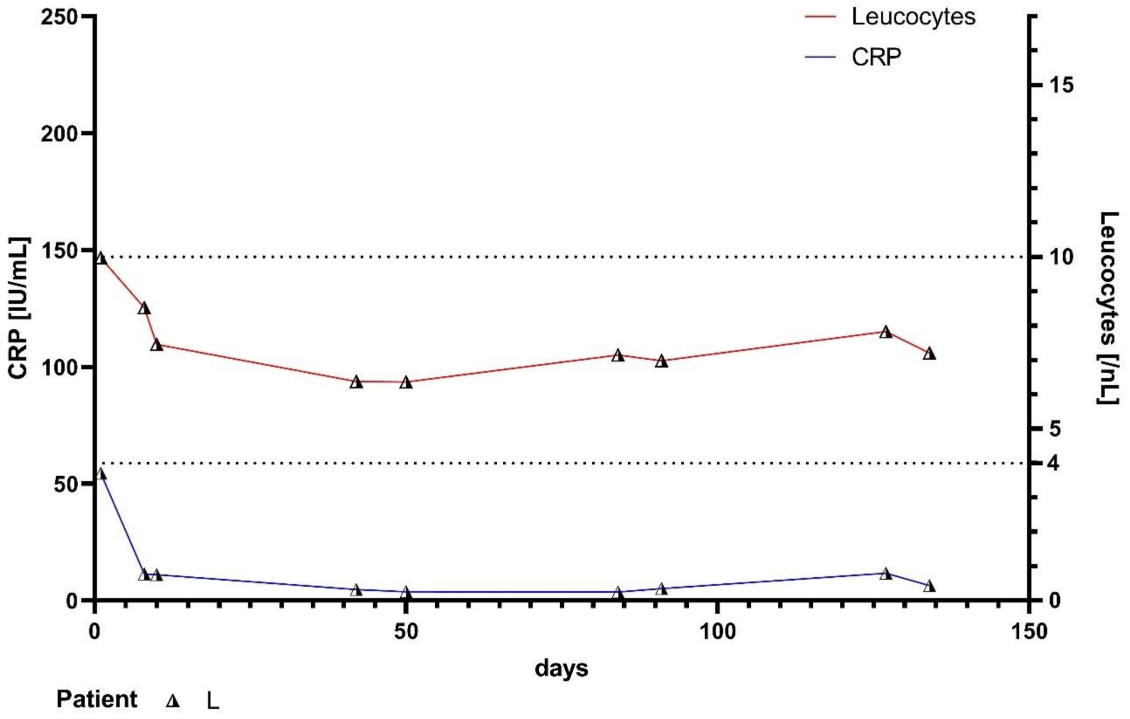

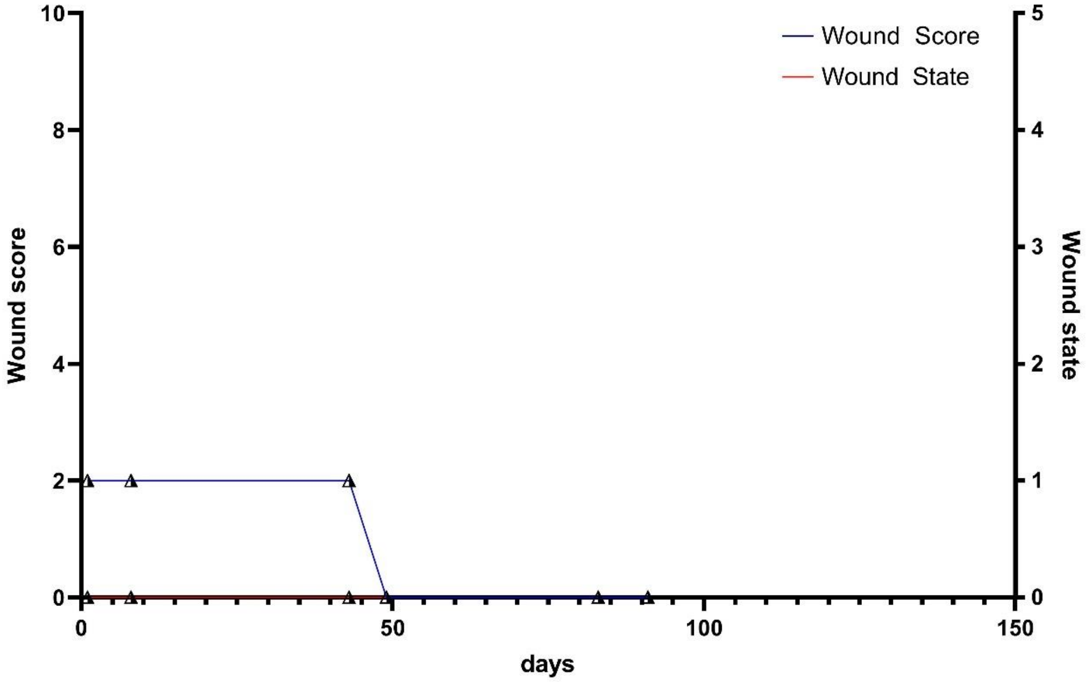

Fig. SI23: C-reactive protein, leucocytes and wound score and wound state according to the adjusted DESTINE-criteria of Patient L. **CRP:** C-reactive protein

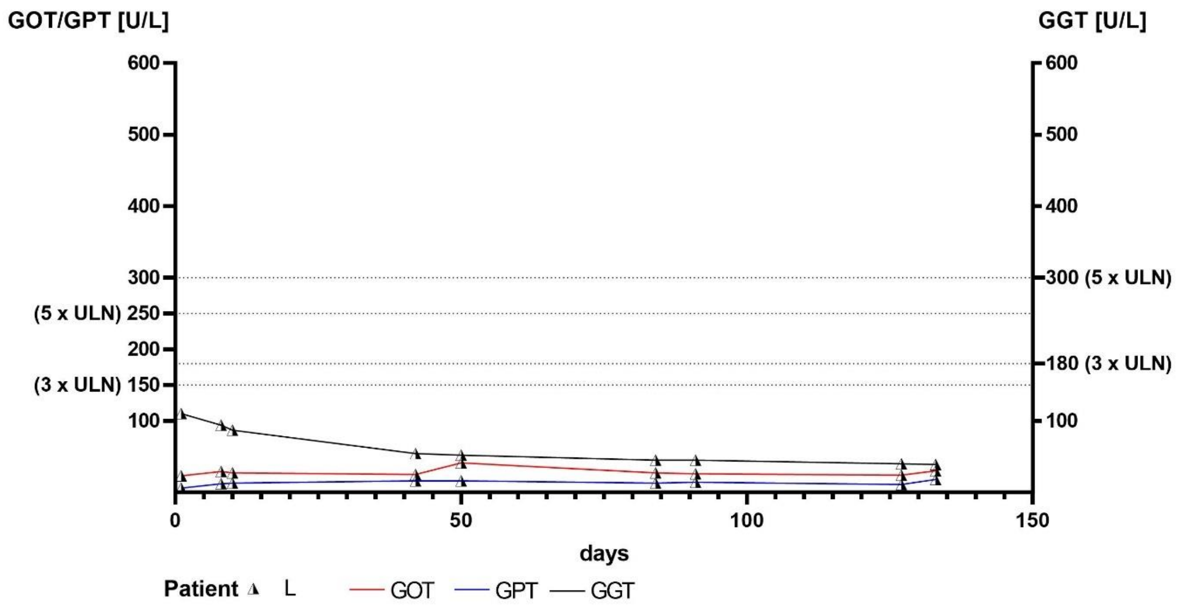

Fig. SI24: GOT, GPT, and GGT of patient L.
GOT: Glutamate-oxalacetate transferase | **GPT:** Glutamate-pyruvate transferase| **GGT:** Gamma-glutamine transferase | **ULN:** Upper limit of normal |

Patient M

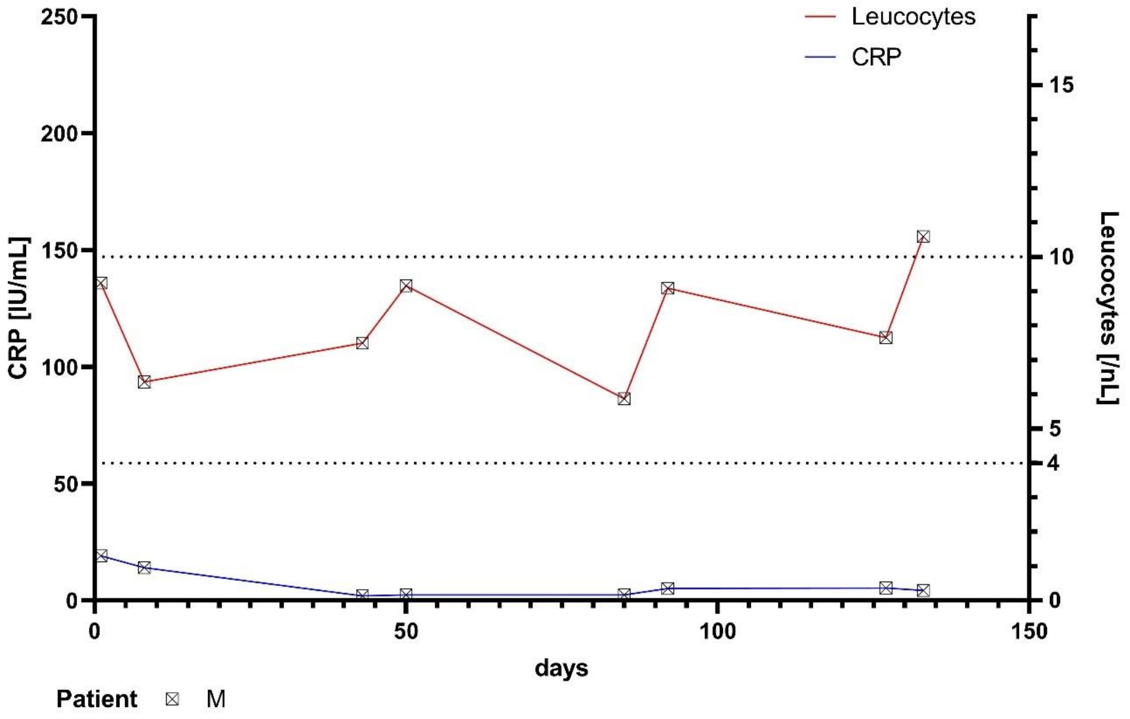

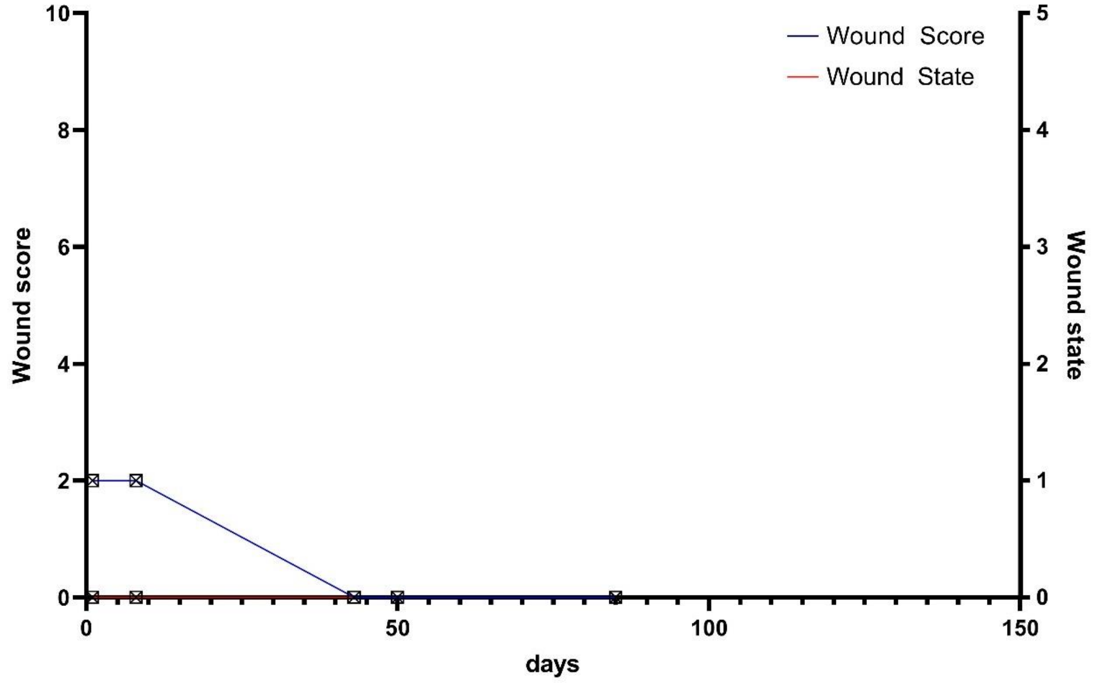

Fig. SI25: C-reactive protein, leucocytes and wound score and wound state according to the adjusted DESTINE-criteria of Patient M. **CRP:** C-reactive protein

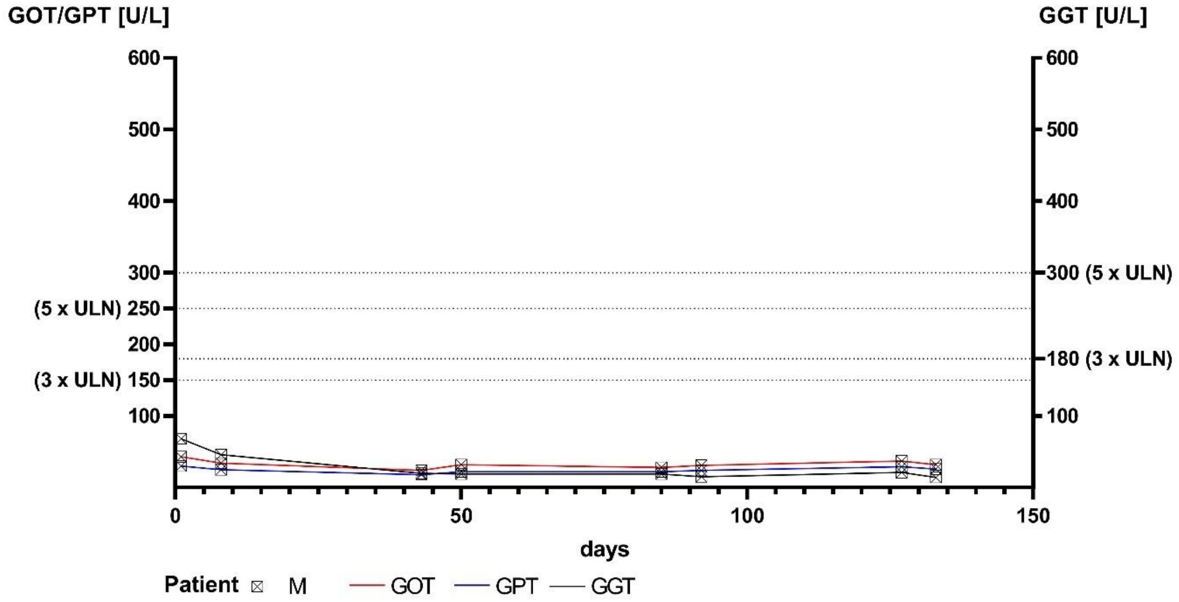

Fig. SI26: GOT, GPT, and GGT of patient M.
GOT: Glutamate-oxalacetate transferase | **GPT:** Glutamate-pyruvate transferase| **GGT:** Gamma-glutamine transferase | **ULN:** Upper limit of normal |

Tables

Table SI1: Diagnoses and comorbidities of the included patients.

Patient	Diagnoses
**A**	LVAD implantation<br>non-ischemic cardiomyopathy<br>arterial hypertension<br>type 2 diabetes mellitus<br>obesity
**B**	LVAD implantation<br>non-ischemic cardiomyopathy following recurrent perimyocarditis<br>severe mitral valve regurgitation<br>reactive arthritis<br>arterial hypertension<br>type 2 diabetes mellitus<br>hypercholesterolemia
**C**	LVAD implantation<br>non-ischemic cardiomyopathy following myocarditis<br>coronary artery disease<br>severe mitral valve regurgitation with MitraClip implantation
**D**	LVAD implantation<br>non-ischemic cardiomyopathy<br>aortic valve replacement (biologic valve) due to moderate regurgitation<br>severe mitral valve reguritation with MitraClip implantation<br>atrial flutter<br>recurrent ventricular tachycardia<br>parathyroid adenoma<br>hypothyreoidism
**E**	LVAD implantation<br>non-ischemic cardiomyopathy<br>cardiogenic shock with multiorgan failure<br>atrial fibrillation<br>severe mitral valve regurgitation
**F**	LVAD implantation<br>ischemic cardiomyopathy<br>Status post coronary artery bypass grafting<br>stent implantation due to LVAD outflow graft stenosis<br>atrial fibrillation<br>MitraClip implantation<br>chronic obstructive pulmonary disease<br>obstructive sleep apnoea<br>pulmonary hypertension<br>chronic renal failure<br>hypothyroidism<br>type 2 diabetes mellitus
**G**	LVAD implantation<br>non-ischemic cardiomyopathy<br>cardiogenic shock with multiorgan failure status post cardiopulmonary resuscitation due to electrical storm

	stellate ganglion block and stereotactic body radiation therapy due to recurrent ventricular tachycardia<br>prostate carcinoma<br>chronic obstructive pulmonary disease<br>chronic kidney disease<br>arterial hypertension<br>hyperlipidaemia<br>type 2 diabetes mellitus
**H**	LVAD implantation<br>non-ischemic cardiomyopathy<br>MitraClip implantation<br>pulmonary hypertension<br>type 2 diabetes mellitus<br>hepatomegaly<br>splenomegaly<br>chronic renal failure
**I**	LVAD implantation<br>non-ischemic cardiomyopathy following myocarditis<br>atrial fibrillation
**J**	LVAD implantation<br>hypertrophic cardiomyopathy<br>cardiogenic shock mit multi organ failure<br>atrial fibrillation<br>hepatomegaly
**K**	LVAD implantation<br>non-ischemic cardiomyopathy following myocarditis<br>stent implantation due to stenosis in LVAD outflow graft<br>severe mitral valve regurgitation<br>tricuspidal valve reconstruction<br>fatty liver disease
**L**	LVAD implantation<br>non-ischemic cardiomyopathy<br>tricuspidal valve reconstruction<br>chronic kidney disease<br>atrial fibrillation<br>cirrhose cardiaque
**M**	LVAD implantation<br>non-ischemic cardiomyopathy following myocarditis<br>severe tricuspid regurgitation<br>pulmonary hypertension<br>multiple sclerosis
**Abbreviations: LVAD:** Left ventricular assist device	

Table SI2: Individual cumulative hospitalization days of all patients before and under dalbavancin

Patient	Before Dalbavancin			Under Dalbavancin		
	Hospitalization for DLI (n)	Hospitalization for BSI (n)	In-hospital days (n)	Hospitalization for DLI (n)	Hospitalization for BSI (n)	In-hospital days (n)
**A**	1	1	58	0	0	0
**B**	0	0	0	1	0	75
**C**	5	0	91	0	0	0
**D**	2	0	56	0	0	0
**E**	1	2	72	0	0	0
**F**	4	0	156	0	0	0
**G**	1	0	31	0	0	0
**H**	4	0	88	0	0	0
**I**	1	0	36	1	0	72
**J**	1	1	87	0	0	0
**K**	2	0	76	0	0	0
**L**	1	0	80	0	0	0
**M**	1	1	29	0	0	0
Abbreviations: **DLI**: Driveline infection | **BSI**: Blood stream infection						

Table SI3: Microbiological results of the included patients under dalbavancin therapy.

Patient	days after first application	driveline	MDRO and MSSA screening
**A**	36	*Staphylococcus capitis*	
	58	*Corynebacterium simulans*	
	99	*Corynebacterium simulans, Serratia marcescens*	
	126	*S. aureus*	MSSA (nose)
	128	*S. aureus, S. marcescens*	
	182	*S. marcescens, Enterococcus faecium*	
		Corynebacterium tuberculostearicum	
	260	*Dermabacter hominis*	
		C. tuberculostearicum, D. hominis	
	338	*C. tuberculostearicum, D. hominis*	
	422	*D. hominis*	
	526	*D. hominis, Staphylococcus epidermidis*	
	604	*S. epidermidis, Schaalia turicensis*	
	694		
	785		
**B**	0	*S. aureus*	MSSA (rectal)
	7	*S. aureus*	
	58	*S. aureus*	
	483	*S. epidermidis*	
	490	*S. epidermidis*	
	525	*S. epidermidis*	
	534	*S. epidermidis*	
	567	*Corynebacterium species*	
	575	*Corynebacterium jeikeium*	
	609	*S. epidermidis, C. jeikeium*	
	652	*C. jeikeium*	
	658	*Citrobacter koseri, Staphylococcus haemolyticus, Enterococcus avium, C. tuberculostearicum, Bacteroides vulgatus, Bacteroides univformis*	
	693	*C. jeikeium*	
	735	*C. jeikeium*	
	777	*C. jeikeium*	
**C**	92		MSSA (nose)
**D**	471	*S. haemolyticus*	
**E**	55	*Staphylococcus saprophyticus*	
	76	*Pantoea agglomerans*	
	146	*Proteus mirabilis, Escherichia coli, E. faecium, Bacteroides species*	
**F**	84		MSSA (nose)
**G**	351	*S. epidermidis*	
	386	*S. epidermidis*	
	393	*Staphylococcus ludgunenesis, S. epidermidis*	
	405	*S. lugdunensis*	

	413	*S. lugdunensis*	
**H**	19<br>43<br>51<br>87<br>91<br>130	 *Klebsiella pneumoniae*<br>*K. pneumoniae*<br>*S. haemolyticus, K. pneumoniae*<br>*S. haemolyticus*<br>*S. epidermidis*	VRE (rectal)
**I**	0<br>48<br>63<br>105<br>113<br>147<br>189<br>238<br>261<br>271<br>279<br><br>283<br>302<br>306<br>321	*S. aureus*<br>*S. aureus*<br><br>*S. aureus*<br>*S. aureus, S. epidermidis*<br>*S. aureus*<br>*S. aureus*<br>*S. aureus*<br>*S. aureus*<br>*S. aureus, S. epidermidis*<br>*S. aureus, S. haemolyticus, S.*<br>*epidermidis*<br>*S. haemolyticus*<br>*S. epidermidis, S. haemolyticus*<br>*S. haemolyticus*<br>*S. haemolyticus, S. epidermidis*	MSSA (nose)<br>MSSA (nose)<br>MSSA (nose)
**J**	42	*S. haemolyticus, S. epidermidis*	
**K**	0<br>8<br>43	*S. haemolyticus, E. faecium*<br><br>*S. haemolyticus*	VRE (rectal)<br>VRE (rectal)
**L**	7<br>41	*S. haemolyticus*	VRE (rectal)<br>VRE (rectal)
**M**		none	
**Abbreviations: MDRO:** Multidrug resistant organisms **| MSSA:** Methicillin sensible *S. aureus* **| VRE:** Vancomycin-resistant *Enterococcus* spp.			

Renal function of the individual patients

Patient A

Tab. SI4: Renal function of patient A under dalbavancin therapy

Day of dalbavancin treatment	eGFR according to CKP-EPI (ml/min/1.73m²)	KDIGO-stage
1	76.1	G2
7	98.1	G1
36	92.4	G1
58	86.3	G2

Abbreviations: CKD-EPI: Chronic kidney disease epidemiology collaboration | **eGFR:** Estimated glomerular filtration rate | **KDIGO**: Kidney disease improving global outcomes

Patient B

Tab.SI5: Renal function of patient B under dalbavancin therapy

Day of dalbavancin treatment	eGFR according to CKP-EPI (ml/min/1.73m²)	KDIGO-stage
1	94.3	G1
7	92.9	G1
51	73.2	G2
141	94.6	G1
162	81.7	G2
190	87.1	G2
240	66	G2
289	68.1	G2
296	64.6	G2
338	68.1	G2
345	67.3	G2
387	69.5	G2
394	64	G2
436	76.1	G2
443	68.8	G2
483	76.1	G2
490	72.7	G2
525	77.3	G2
534	72.2	G2
567	70.6	G2
574	72.2	G2
609	72.2	G2
616	76.4	G2
652	64.2	G2
658	69.8	G2
693	71.4	G2
700	68.3	G2
735	66.2	G2
743	72.2	G2
819	73.8	G2
827	66.2	G2

861	78.6	G2
868	77.7	G2

Abbreviations: CKD-EPI: Chronic kidney disease epidemiology collaboration | **eGFR:** Estimated glomerular filtration rate | **KDIGO**: Kidney disease improving global outcomes

Patient C

Tab.SI6: Renal function of patient C under dalbavancin therapy

Day of dalbavancin treatment	eGFR according to CKP-EPI (ml/min/1.73m²)	KDIGO-stage
1	108.1	G1
7	107.5	G1
15	103.8	G1
43	101.4	G1

Abbreviations: CKD-EPI: Chronic kidney disease epidemiology collaboration | **eGFR:** Estimated glomerular filtration rate | **KDIGO**: Kidney disease improving global outcomes

Patient D

Tab. SI7: Renal function of patient D under dalbavancin therapy

Day of dalbavancin treatment	eGFR according to CKP-EPI (ml/min/1.73m²)	KDIGO-stage
7	66.1	G2
43	54.4	G3a
55	52.6	G3a
55	50.9	G3a
90	49	G3a
97	48.2	G3a
141	55.5	G3a
147	66.3	G2
189	49.4	G3a
196	49.8	G3a
239	48.6	G3a
246	60.1	G2
294	30	G4
303	44.4	G3b
337	57	G3a
343	40.6	G3b
378	60.7	G2
387	65.6	G2
420	58	G3a
427	47.1	G3a
462	63.1	G2
471	63.7	G2
504	57.1	G3a
511	53.7	G3a
546	61.4	G2
554	53.7	G3a
588	60.9	G2
595	48.3	G3a

630	54.6	G3a
638	49	G3a
672	55.6	G3a
679	38.5	G3b
715	59.7	G3a
721	56.1	G3a
755	46.5	G3a
763	55.1	G3a

Abbreviations: CKD-EPI: Chronic kidney disease epidemiology collaboration | **eGFR:** Estimated glomerular filtration rate | **KDIGO**: Kidney disease improving global outcomes

Patient E

Tab.SI8: Renal function of patient E under dalbavancin therapy.

Day of dalbavancin treatment	eGFR according to CKP-EPI (ml/min/1.73m²)	KDIGO-stage
1	91.9	G1
7	96.3	G1
21	94.5	G1
47	81	G2
54	81	G2
76	77.7	G2
96	75.9	G2
103	82.4	G2
145	71.8	G2
153	88.9	G2
194	82.4	G2
201	88.9	G2
244	88.9	G2
250	73.4	G2
285	81.4	G2
292	92.5	G1
327	88.9	G2
335	86.7	G2
369	88.9	G2
385	85.6	G2
392	85.6	G2
425	84.5	G2
432	89.5	G2
454	90.6	G1
467	94.5	G1
474	94.5	G1
510	94.5	G1
516	87.2	G2
553	94.5	G1
558	93.6	G1
594	85	G2
600	95	G1
635	85	G2
642	88.3	G2

678	88.3	G2
684	95.4	G1
719	81.9	G2
726	89.5	G2

Abbreviations: CKD-EPI: Chronic kidney disease epidemiology collaboration | **eGFR:** Estimated glomerular filtration rate | **KDIGO**: Kidney disease improving global outcomes.

Patient F

Tab.SI9: Renal function of patient F under dalbavancin therapy.

Day of dalbavancin treatment	eGFR according to CKP-EPI (ml/min/1.73m²)	KDIGO-stage
1	30.4	G3b
7	33.1	G3b
28	32.1	G3b
52	26.7	G4
59	35	G3b
64	30.5	G3b
107	36.4	G3b
114	31.9	G3b
160	34.6	G3b
168	32.1	G3b
202	32.1	G3b
209	33.7	G3b

Abbreviations: CKD-EPI: Chronic kidney disease epidemiology collaboration | **eGFR:** Estimated glomerular filtration rate | **KDIGO**: Kidney disease improving global outcomes.

Patient G

Tab.SI10: Renal function of patient G under dalbavancin therapy.

Day of dalbavancin treatment	eGFR according to CKP-EPI (ml/min/1.73m²)	KDIGO-stage
1	41.7	G3b
7	42.9	G3b
50	36.5	G3b
56	31	G3b
91	29.4	G4
98	27.6	G4
133	32.1	G3b
141	29.2	G4
175	29.8	G4
182	27.5	G4
217	34.5	G3b
225	34.5	G3b
259	30	G4
266	35.8	G3b
301	33.1	G3b
310	33.9	G3b
343	32	G3b

350	30.8	G3b
385	34.9	G3b
393	33.1	G3b

Abbreviations: CKD-EPI: Chronic kidney disease epidemiology collaboration | **eGFR:** Estimated glomerular filtration rate | **KDIGO**: Kidney disease improving global outcomes.

Patient H

Tab.SI11: Renal function of patient H under dalbavancin therapy.

Day of dalbavancin treatment	eGFR according to CKP-EPI (ml/min/1.73m²)	KDIGO-stage
1	81.4	G2
6	69.1	G2
19	77.7	G2
20	69.8	G2
21	70.5	G2
22	65.7	G2
23	62	G2
24	64.4	G2
26	65.7	G2
27	69.8	G2
28	61.4	G2
29	62	G2
30	64.4	G2
31	60.9	G2
32	57.1	G3a
33	53.4	G3a
35	55.7	G3a
37	58.7	G3a
39	54.3	G3a
41	52.1	G3a
43	53.4	G3a
45	62	G2
47	58.7	G3a
49	57.1	G3a
51	59.2	G3a
53	60.3	G2
55	55.7	G3a
58	58.2	G3a
60	55.2	G3a
62	57.1	G3a
64	62.6	G2
66	52.5	G3a
67	59.2	G3a
69	51.3	G3a
71	53.8	G3a
73	54.7	G3a
75	56.7	G3a
77	45.4	G3a
79	46.4	G3a

81	51.7	G3a
83	51.7	G3a
85	52.1	G3a
87	55.2	G3a
89	56.7	G3a
91	58.7	G3a
93	42.9	G3b
95	56.7	G3a
97	57.1	G3a
99	57.1	G3a
101	60.9	G2
106	52.5	G3a
108	56.7	G3a
110	54.7	G3a
112	52.1	G3a
114	51.3	G3a
115	52.9	G3a
117	51.7	G3a
118	51.7	G3a
119	50.8	G3a
121	50.4	G3a
123	48.9	G3a
125	54.3	G3a
127	52.1	G3a
129	53.8	G3a
131	48.9	G3a

Abbreviations: CKD-EPI: Chronic kidney disease epidemiology collaboration | **eGFR:** Estimated glomerular filtration rate | **KDIGO**: Kidney disease improving global outcomes.

Patient I

Tab.SI12: Renal function of patient I under dalbavancin therapy.

Day of dalbavancin treatment	eGFR according to CKP-EPI (ml/min/1.73m²)	KDIGO-stage
1	65.3	G2
7	60.9	G2
41	58.1	G3a
63	64.8	G2
70	66.2	G2
105	73.8	G2
113	69	G2
147	70.6	G2
154	61.7	G2
189	72.2	G2
197	64.2	G2
231	64.2	G2
238	69	G2
334	58.8	G3a
342	69	G2
357	68.3	G2

376	66.2	G2
383	72.2	G2
420	69.3	G2

Abbreviations: CKD-EPI: Chronic kidney disease epidemiology collaboration | **eGFR:** Estimated glomerular filtration rate | **KDIGO**: Kidney disease improving global outcomes.

Patient J

Tab. SI13: Renal function of patient J under dalbavancin therapy.

Day of dalbavancin treatment	eGFR according to CKP-EPI (ml/min/1.73m²)	KDIGO-stage
1	108.5	G1
6	103.7	G1
42	88.4	G2
49	93.7	G1
84	76	G2
91	77.5	G2
126	79.2	G2
133	74.4	G2
168	75.2	G2
175	80	G2
203	66.4	G2
209	68.9	G2
217	74.4	G2
252	68.3	G2

Abbreviations: CKD-EPI: Chronic kidney disease epidemiology collaboration | **eGFR:** Estimated glomerular filtration rate | **KDIGO**: Kidney disease improving global outcomes.

Patient K

Tab.SI14: Renal function of patient K under dalbavancin therapy.

Day of dalbavancin treatment	eGFR according to CKP-EPI (ml/min/1.73m²)	KDIGO-stage
1	52.3	G3a
8	55.8	G3a
21	43.5	G3b
43	62	G2
50	60.4	G2
85	57.8	G3a
91	49.2	G3a
127	49.9	G3a
134	63.2	G2
168	51.5	G3a
176	43.2	G3b
212	59.3	G3a

Abbreviations: CKD-EPI: Chronic kidney disease epidemiology collaboration | **eGFR:** Estimated glomerular filtration rate | **KDIGO**: Kidney disease improving global outcomes.

Patient L

Tab.SI15: Renal function of patient L under dalbavancin therapy.

Day of dalbavancin treatment	eGFR according to CKP-EPI (ml/min/1.73m²)	KDIGO-stage
1	73.6	G2
7	75.3	G2
41	47.9	G3a
49	35.7	G3b
83	47.1	G3a
90	46.8	G3a
126	36.6	G3b
133	49	G3a
167	45.8	G3a

Abbreviations: CKD-EPI: Chronic kidney disease epidemiology collaboration | **eGFR:** Estimated glomerular filtration rate | **KDIGO**: Kidney disease improving global outcomes.

Patient M

Tab.SI16: Renal function of patient M under dalbavancin therapy.

Day of dalbavancin treatment	eGFR according to CKP-EPI (ml/min/1.73m²)	KDIGO-stage
1	124.10	G1
7	121.40	G1
42	122.70	G1
49	123.40	G1
84	120.50	G1
91	121.20	G1
126	115.00	G1
132	124.00	G1

Abbreviations: CKD-EPI: Chronic kidney disease epidemiology collaboration | **eGFR:** Estimated glomerular filtration rate | **KDIGO**: Kidney disease improving global outcomes.
